# HIV-1 Envelope Subregion Length Variation during Disease Progression

**DOI:** 10.1371/journal.ppat.1001228

**Published:** 2010-12-16

**Authors:** Marcel E. Curlin, Rafael Zioni, Stephen E. Hawes, Yi Liu, Wenjie Deng, Geoffrey S. Gottlieb, Tuofu Zhu, James I. Mullins

**Affiliations:** 1 Department of Medicine, University of Washington School of Medicine, Seattle, Washington, United States of America; 2 Department of Laboratory Medicine, University of Washington School of Medicine, Seattle, Washington, United States of America; 3 Department of Epidemiology, University of Washington School of Public Health, Seattle, Washington, United States of America; 4 Department of Microbiology, University of Washington School of Medicine, Seattle, Washington, United States of America; University of Zurich, Switzerland

## Abstract

The V3 loop of the HIV-1 Env protein is the primary determinant of viral coreceptor usage, whereas the V1V2 loop region is thought to influence coreceptor binding and participate in shielding of neutralization-sensitive regions of the Env glycoprotein gp120 from antibody responses. The functional properties and antigenicity of V1V2 are influenced by changes in amino acid sequence, sequence length and patterns of N-linked glycosylation. However, how these polymorphisms relate to HIV pathogenesis is not fully understood. We examined 5185 HIV-1 gp120 nucleotide sequence fragments and clinical data from 154 individuals (152 were infected with HIV-1 Subtype B). Sequences were aligned, translated, manually edited and separated into V1V2, C2, V3, C3, V4, C4 and V5 subregions. V1-V5 and subregion lengths were calculated, and potential N-linked glycosylation sites (PNLGS) counted. Loop lengths and PNLGS were examined as a function of time since infection, CD4 count, viral load, and calendar year in cross-sectional and longitudinal analyses. V1V2 length and PNLGS increased significantly through chronic infection before declining in late-stage infection. In cross-sectional analyses, V1V2 length also increased by calendar year between 1984 and 2004 in subjects with early and mid-stage illness. Our observations suggest that there is little selection for loop length at the time of transmission; following infection, HIV-1 adapts to host immune responses through increased V1V2 length and/or addition of carbohydrate moieties at N-linked glycosylation sites. V1V2 shortening during early and late-stage infection may reflect ineffective host immunity. Transmission from donors with chronic illness may have caused the modest increase in V1V2 length observed during the course of the pandemic.

## Author Summary

The HIV envelope gene (*env*) encodes viral surface proteins (Env) that are vital to the basic processes used by the virus to infect and cause disease in humans. Adaptations in *env* determine which cells the virus can infect, and permit the virus to avoid elimination by the immune system. *Env* is one of the most variable genes known, and it can change dramatically over time in a single individual. However, Env-host cell interactions are complex and incompletely understood, and changes in this viral protein during infection have not yet been systematically described. We examined a large number of *env* sequences from 154 individuals at various stages of HIV infection but who had never received antiretroviral treatment. We found that the *env* V1V2 region lengthens during chronic infection and becomes more heavily glycosylated. However, these changes partially reverse during late-stage illness, possibly in response to a weakening host immune system. V1V2 lengths are also increasing over time in the epidemic at large, possibly related to the epidemiology of HIV transmission within the subtype B epidemic. These results provide fundamental insights into the biology of HIV.

## Introduction

The gp120 portion of the HIV-1 envelope protein (Env) mediates attachment prior to fusion with the host cell membrane during target cell infection. gp120 has five hypervariable regions (V1–V5) bounded by cysteine residues and separated by four relatively “constant” regions (C1–C4) [1]–[3]. Gp120 is notable for its sequence variation, which may arise through recombination and point mutation, as well as by insertion and deletion of one or more nucleotides. Insertion and deletion events (indels) occur throughout *env* but are maintained through positive selection particularly within the hypervariable loops, which thereby may acquire significant length variation [4], The third hypervariable region is known to encode the primary determinants of coreceptor usage specificity [5]–[7], as well as epitopes recognized by humoral [8], [9] and cellular [10], [11] immune responses. V3 loop sequence variation has been extensively studied, and correlated with changes in host cell range, cytopathogenicity, and disease progression [12]–[14].

The V1V2 region in particular is characterized by a high degree of length polymorphism, sequence variation, and predicted N-linked glycosylation sites (PNLGS) [15]–[20], each of which may affect viral attachment, coreceptor usage and recognition by neutralizing antibodies [20], [21]. Comparison of structural models of gp120 and gp120 bound to CD4 and a chemokine coreceptor have yielded considerable insight into the functional roles played by V1V2 and V3 during viral attachment [22], [23]. In the unbound gp120 conformation, the V2 loop partially obscures V3 and other gp120 residues involved in coreceptor binding. Binding to CD4 induces conformational changes that expose the coreceptor binding site on gp120, including residues from V1V2, V3 and other regions [22], [24].

Numerous studies have suggested that sequence variation in V1V2 influences host cell range and/or syncytium-inducing (SI) phenotype [25]–[31]. For example, Toohey demonstrated that recombinant chimeric clones with a V1V2 region from macrophage-tropic HIV-1 strains replicated efficiently in macrophages, whereas clones with the V1V2 region from lymphotropic strains did not [31]. However, not all studies have been concordant on the role of V1V2 in viral replication kinetics, cell range and transmission [15]–[19], [32]. For example, Pastore showed that sequence changes in V1V2 could rescue otherwise lethal mutations in V3 associated with a change in coreceptor usage [33], and V2 polymorphisms have also been linked with restriction to CCR5 coreceptor usage [16]. In contrast, Wang et al found no relationship between SI phenotype and V1V2 sequence, length, distribution of PNLGS or charge [32].

The V1V2 region also appears to be an important determinant of sensitivity to neutralizing antibodies [34]–[38]. The V1V2 region evolves under positive natural selection *in vivo* [4], [39]–[41], and an inverse relationship between V1–V4 length and neutralization susceptibility has been demonstrated in subtypes A [20], B [34]–[38] and C [42]. Tellingly, laboratory strains lacking V1V2 may still replicate efficiently *in vitro*, but appear to be especially sensitive to antibody neutralization [43], [44]. Consistent with this observation, viral strains with shorter and less glycosylated V1V4 regions have been reported to preferentially replicate in subjects newly infected with HIV-1 subtype C [45] (where presumably an effective neutralizing antibody response has not had time to emerge), and similar observations have been made concerning the V1V2 loop in individuals recently infected by HIV-1 subtype A [19]. However, we and others have not observed this effect in HIV-1 subtype B [19], [46], [47].

Despite these reports, the relationship between V1V2 region length polymorphism and disease progression remains unclear. In two small longitudinal studies, elongation of V1 and V2 was noted in long-term nonprogressors (LTNP), but not within individuals progressing rapidly to AIDS [15]–[19]. In a third study, no clear relationship between V1V2 length variation and disease progression was observed [48]. Lastly, some investigators postulate that V1V2 length changes positively correlate with the pace of disease progression [16], [19], while others have suggested that V1V2 length increase may be a correlate of delayed progression to AIDS [18].

Thus, our understanding of the role of the V1V2 loop in influencing HIV pathogenesis remains incomplete and is challenged by several contradictory observations. To more fully characterize HIV envelope subregion variability and to clarify the associations between subregion length variation, glycosylation, and disease progression, we have comprehensively examined length and glycosylation of each gp120 subregion as a function of clinical parameters in a large collection HIV-1 subtype B infected individuals.

## Methods

### Ethics statement

This study was performed using publicly available data from the Los Alamos database, and previously unpublished experimental data obtained at the University of Washington. Unpublished data were obtained and analyzed with written informed consent of study participants, and approval by the University of Washington Institutional Review Board.

### Patient selection

We analyzed new and published HIV-1 envelope gene sequences and associated clinical data from all available subjects in the Seattle Primary Infection Cohort (PIC) [49], the Multicenter AIDS Cohort Study (MACS) [50], and from the Los Alamos National Laboratories HIV database (HIVDB) (http://www.hiv.lanl.gov/content/hiv-db/mainpage.html) not meeting pre-specified exclusion criteria. Subjects were excluded from this study if younger than 18 years of age or if there was any history of antiretroviral therapy prior to sampling as determined by patient report and clinical records (MACS, PIC) or as indicated in the methods section of published reports (HIVDB), unless otherwise noted. All subjects considered in the cross-sectional and longitudinal analyses were infected with HIV-1 subtype B, except for two subjects infected with HIV-1 subtype A who were included in longitudinal analyses, but were excluded from cross-sectional analyses. (Additional subtypes were considered in analyses of *env* subregion length change during transmission, presented in Text S1, Section 8). Clinical data retrieved included CD4 count, viral load, time since infection, and treatment history. Sequence data were only accepted if directly derived from plasma or PBMC without an intervening step involving viral propagation *in vitro*. In some cases, individual authors were consulted to resolve clinical or methodological ambiguities. Accession numbers for published sequences are provided in Table S1. Gene sequence data used in this study are available at http://mullinslab.microbiol.washington.edu/publications/curlin_2010/.

### Subject groups

Viral gene sequence data were considered in both cross-sectional (Table 1) and longitudinal analyses (Table 2). The cross-sectional dataset included only plasma and PBMC sequences derived from individuals infected with subtype B (see results, and Table 1). Sequences were triaged by author, database identifier and associated clinical data to exclude duplicate entries. To assess the role of stage of illness on loop length variation, subjects were divided into four non-overlapping groups; *group C_x_1* subjects were sampled within two months of the estimated time of infection. *Group C_x_2* subjects were sampled between two months and three years following infection. *Group C_x_3* subjects were sampled at times >3 years post infection. *Group C_x_4* was comprised of all individuals meeting 1993 CDC criteria for AIDS when sampling occurred (generally CD4 count <200/mm^3^), regardless of time since infection.

**Table 1:** Distribution of subjects, samples and sequences in cross-sectional analyses.

Cross-sectional Data									
Group	Subjects	Samples	Sequences						
			V1V2	C2	V3	C3	V4	C4	V5
**TOTAL**	152	453	1922	1275	4407	3616	4406	4405	4407
**MACS**	27	227	682	682	2567	2568	2567	2569	2569
**PIC**	43	78	541	390	846	845	845	845	847
**LANL**	82	148	699	203	994	203	994	991	991
**Sequences by category**									
**PBMC**	62	225	385	65	2193	1675	2193	2193	2193
**Plasma**	90	228	1537	1210	2214	1941	2213	2212	2214
**Asia**	11	16	176	0	0	0	0	0	0
**North America**	111	362	1585	1207	3725	3548	3724	3726	3728
**South America**	5	5	5	5	5	5	5	5	5
**Western Europe**	25	70	156	63	677	63	677	674	674
**Stage 1**	41	42	418	365	383	383	383	384	384
**Stage 2**	40	146	872	741	1540	1483	1539	1540	1542
**Stage 3**	22	156	220	94	1586	1029	1586	1583	1583
**Stage 4**	11	63	170	35	631	631	631	631	631
**Unknown Stage**	38	46	242	40	267	90	267	267	267

Number of subjects, samples, and available coding sequences by cohort, gene region, anatomical site, geographic location, and stage of illness.

**Table 2:** Distribution of subjects, samples and sequences in longitudinal analyses.

Longitudinal Data									
Group	Subjects	Samples	Sequences						
			V1V2	C2	V3	C3	V4	C4	V5
**TOTAL**	22	83	807	30	155	155	155	155	155
**PIC**	1	15	180	30	155	155	155	155	155
**LANL**	21	68	627	0	0	0	0	0	0
**Sequences by category**									
**Culture**	NA	1	33	0	0	0	0	0	0
**Cervical Swab**	NA	7	37	0	0	0	0	0	0
**PBMC**	N/A	43	397	0	0	0	0	0	0
**Plasma**	N/A	32	340	30	155	155	155	155	155
**Subtype A**	2	26	172	0	0	0	0	0	0
**Subtype B**	20	57	635	30	155	155	155	155	155
**Asia**	4	9	102	0	0	0	0	0	0
**East Africa**	2	26	172	0	0	0	0	0	0
**North America**	12	40	440	30	155	155	155	155	155
**Western Europe**	4	8	93	0	0	0	0	0	0
**Stage 1**	NA	5	63	10	23	23	23	23	23
**Stage 2**	NA	12	140	10	111	111	111	111	111
**Stage 3**	NA	10	111	10	21	21	21	21	21
**Stage 4**	NA	9	86	0	0	0	0	0	0
**Unknown Stage**	10	47	407	0	0	0	0	0	0

Number of subjects, samples, and available coding sequences by cohort, gene region, anatomical site, HIV subtype, geographic location, and stage of illness.

The longitudinal dataset was derived from 20 subjects infected with subtype B and 2 individuals infected with subtype A, from the PIC cohort and from previous reports [18], [51]–[55], in whom data were available from two or more timepoints (see results, and Table 2). All intra-individual longitudinal comparisons were made between sequences obtained from the same compartment (e.g., plasma *vs.* plasma). Individuals partitioned into group *L1* (N = 15) did not meet criteria for AIDS at any time prior to the final sample (median follow-up 3.25 years, range 1 to 20.8 years), whereas subjects in group *L2* (N = 7) were reported to have an AIDS-defining illness or peripheral CD4 count <200/mm^3^ between the first and second samples (median follow-up 2.75 years, range 2 to 4 years).

### Nucleic acid isolation, cloning and sequencing

Sequences from the PIC and MACS cohorts (Tables 1 & 2) were obtained from plasma or PBMC by standard methods [56], [57], using safeguards to prevent contamination and template resampling [58]. Briefly, PCR amplification was performed using Taq polymerase (Bioline) with primers ED3 and BH2 [59] (first round) followed by ED5 and DR7 (second round) [60]. PCR products were cloned into a TA TOPO vector (Invitrogen) and selected colonies sequenced under contract using Big Dye dye-terminator protocols. Genbank accession numbers pending submission.

### Sequence analysis

Deduced amino acid sequences were aligned using ClustalW [61] and divided into seven subregions; V1V2 (HXB2 nucleotide positions 6615–6812), C2 (HXB2 6813-7109), V3 (HXB2 7110–7217), C3 (HXB2 7218–7376), V4 (HXB2 7377–7478), C4 (HXB2 7479–7556), and V5 (HXB2 7557–7637). Alignments were manually edited and subregion lengths were counted using MacClade. PNLGS were counted using NetNGlyc.1 (http://www.cbs.dtu.dk/services/NetNGlyc/). Coreceptor usage (CCR5 *vs*. CXCR4 tropism) was predicted for all available subtype B V3 loop sequences, using the Position-Specific Substitution Method (PSSM) [62], Geno2pheno [63] and two other machine learning algorithms [64], [65] (hereafter denoted PSSM, G2P, PGRC and BMLC, respectively). For G2P coreceptor usage predictions, we selected the standard 10% false positivity threshold, and PGRC predictions were based on the support vector machine (SVR) user option. Estimated time since infection was calculated for all data entries. When time was reported as time since onset of symptoms or time post seroconversion (SC), symptoms and seroconversion were assumed to occur at 14 days and 42 days after infection, respectively [66], [67]. Date of seroconversion was assumed to occur at the midpoint between most recent negative serological test and first reported positive test, unless additional information was available.

### Statistical analysis

For cross-sectional analyses, univariate and multivariate regressions were conducted assessing subregion lengths and number of glycosylation sites as a function of time since infection, stage of disease, CD4 count, HIV viral load, adjusting for sample source (plasma *vs.* PBMC), and date of sampling (calendar year). In regression analyses, to allow direct comparisons of the effect of each variable on V1V2 length and/or glycosylation, we compared β values (i.e., regression coefficients scaled such that each variable is equivalent to having a mean value of 0 and a standard deviation of 1). Generalized estimating equations (GEE) were utilized to account for non-independence of data points [68]–[70], and an exchangeable correlation structure was assumed. This method adjusts for the correlation of multiple sequences nested within a sample as well as multiple samples per patient. As an additional means of verifying that analysis outcomes were not influenced by data linkage, regression analyses were performed on replicate data subsets reconstituted from the original data by random resampling, including analyses on 100 data subsets each obtained by using one randomly selected sequence from each individual (See Text S1 section S2). To ensure that results were not unduly influenced by outlying sequences with extremely short or long loop lengths, analyses were repeated after excluding sequences representing the shortest 5% and longest 5% of the V1V2 loops in the dataset. For the longitudinal dataset, multivariate linear regressions were conducted assessing V1V2 length and number of glycosylation sites as a function of time since infection within a person, and the mean rate of change per year was estimated. Statistical analyses were performed using SAS version 9.1 (SAS Institute, Cary, NC).

## Results

### Sequence data

We obtained 5185 partial length HIV-1 env gene sequences for cross-sectional and longitudinal analysis by the methods described above (Tables 1 & 2). Sequences were isolated from 475 samples obtained from 154 individuals, including 27 from the MACS, 43 from the Seattle PIC and 84 from the HIVDB. Study subjects resided in North America (N = 116), Western Europe (N = 25), East Africa (N = 2), and Asia (N = 11), contributed a median of 14 sequences (range 1–287) and included persons in stages 1 (N = 41), 2 (N = 62), 3 (N = 40), and 4 (N = 27) of infection (note that some subjects contributing to the longitudinal analysis were included at more than one stage of infection). Sequences were derived from plasma (N = 2495), PBMC (N = 2620) and other sites (N = 70). Sequences were of subtype B (N = 5013) and subtype A (N = 172). All subtype A sequences and sequences derived from sites other than blood were excluded from cross-sectional analyses, but were considered as special cases under longitudinal analyses (sequence data available at: *webaddress pending acceptance*).

### Cross-sectional analyses

#### Variation in sequence length and glycosylation

**Figure 1:**
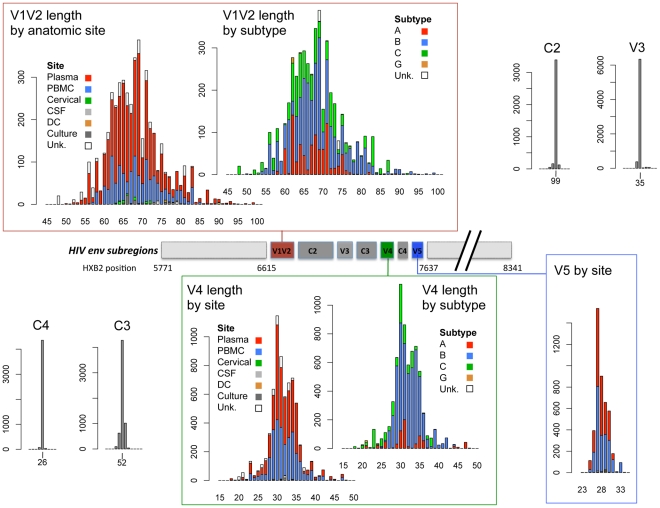
Schematic diagram of HIV-1 *env* subregions (center bar) and distribution of subregion loop lengths (surrounding bar graphs). The center bar depicts the linear arrangement of subregions V1V2 through V5 within the HIV Env gp120 protein. The amino acid length distribution of each subregion is shown in the linked bar graphs, including sequences in the cross-sectional dataset, the longitudinal dataset and the transmission data described in Text S1. Length distributions in V1V2 and V4 data are shown by isolation site (PBMC  =  blue bars, plasma  =  red bars, cervical cells  =  green bars, CSF  =  light gray bars, dendritic cells  =  orange bars, cell culture  =  dark gray bars, and cells from unknown anatomic compartments represented by open bars), and by subtype (subtype B  =  blue bars, subtype A  =  red bars, subtype C  =  green bars, subtype G  =  orange bars, untyped sequences  =  open bars). V5 sequences were all of subtype B. X-axis: sequence length (amino acids); Y-axis: number of sequences.

The V1V2, V4 and V5 hypervariable regions displayed heterogeneity in lengths up to approximately 2-fold in the 152 individuals examined. V1V2 was the most variable region, with loop lengths ranging from 50 to 99 amino acids (mean = 68), while V4 and V5 loop lengths ranged from 19 to 44 (mean = 32), and 14 to 36 (mean = 28) amino acids, respectively. In contrast, the V3 loop and the C2, C3 and C4 regions showed relatively little length variation (Figure 1). The subregions with the greatest number of potential glycosylation sites were V1V2 (mean 6 sites, range 0–12), C2 (mean 5, range 3–8) and V4 (mean 5, range 1–7). V3, C3 and V5 were more modestly glycosylated (mean = 1, 3, and 2, respectively, with a maximum of 5 glycosylated sites), whereas C4 rarely contained potential glycosylation sites (1 site was found in 8 of 4403 sequences).

#### Relationship between V1V2 loop length, sample features and clinical factors – univariate analyses

**Figure 2:**
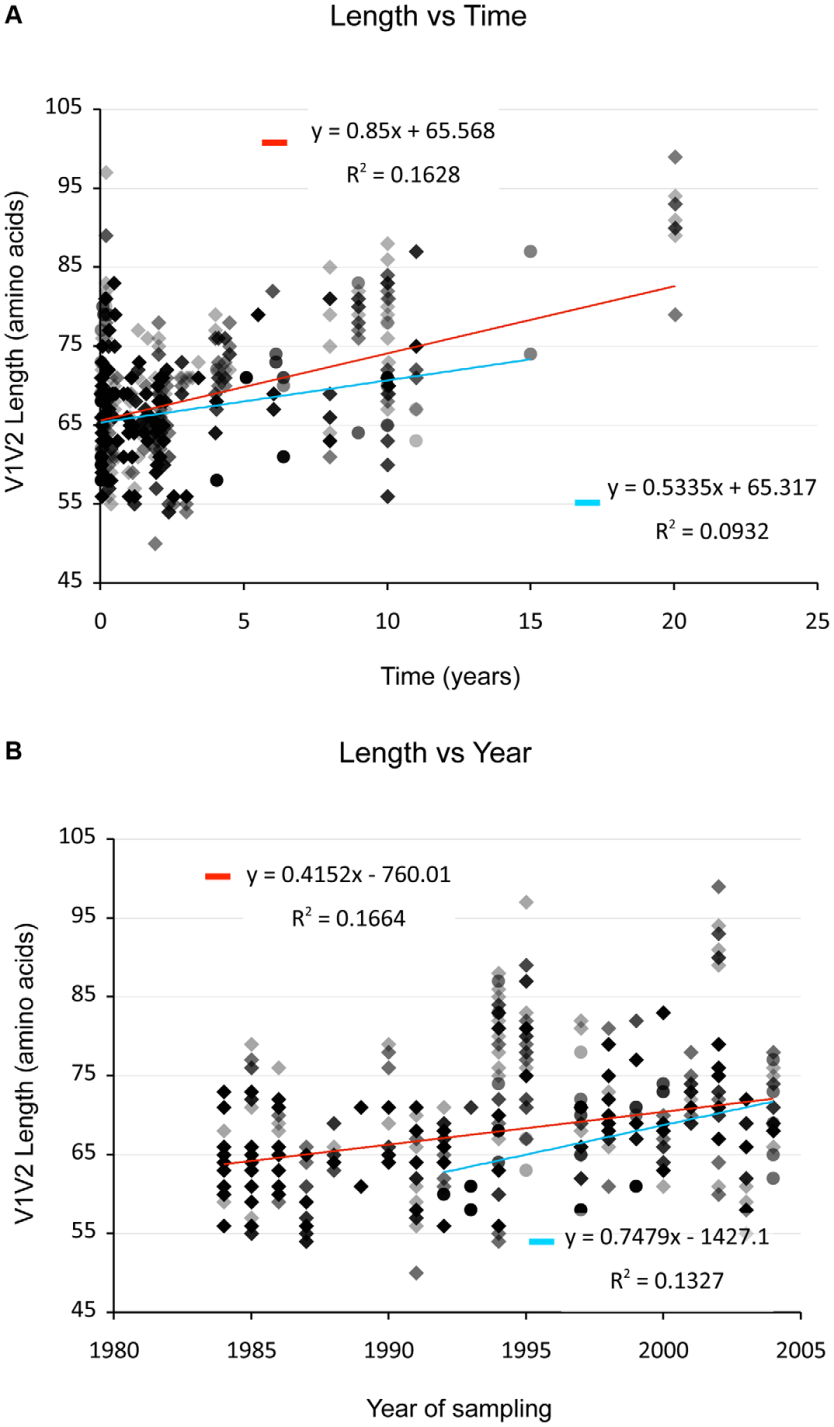
V1V2 length *vs*. time since infection (upper panel) and vs. year of sampling (lower panel). Lengths are indicated in amino acids. Overlapping data points appear as darker symbols. Sequences from plasma are represented by diamonds and sequences from PBMC are represented by circles. Regression coefficients and coefficients of determination are shown for univariate linear regression, for plasma (red line) and PBMC (blue line).

**Figure 3:**
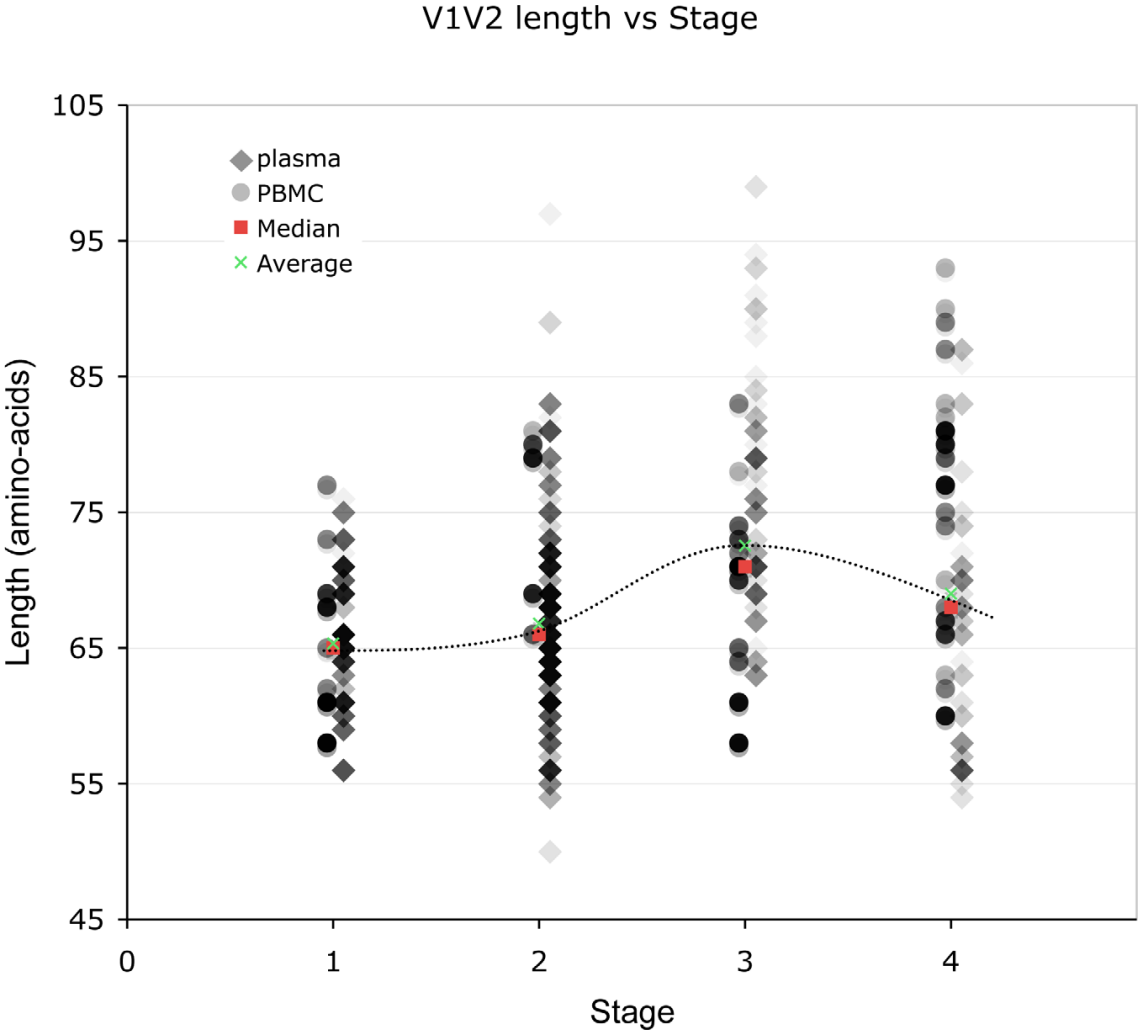
Correlation between stage of illness and V1V2 length. Lengths are indicated in amino acids. Sequences from plasma are represented by diamonds and sequences from PBMC are represented by circles. Overlapping data points appear as darker symbols. Quartiles and median values are indicated by horizontal line segments. Stage 1, 2, and 3 subjects were sampled within two months, between two months and three years, and at times >3 years post infection, respectively. Stage 4 subjects were comprised of all individuals meeting 1993 CDC criteria for AIDS when sampling occurred, regardless of time since infection.

(And see Text S1, sections S1, S3, S6, and Figures S1, S2 and S12.) We examined V1V2 loop lengths as a function of year of sampling and specimen type (plasma *vs.* PBMC). In separate univariate GEE analyses, V1V2 length increased with calendar year of sampling (β = 1.62 increase in V1V2 length per year; p = 0.003, Figure 2, lower panel) and trended towards greater length in PBMC, though not significantly (β = 1.70 for PBMC compared to plasma; p = 0.11). We then examined individual subregion lengths as a function of time since infection, clinical stage, CD4 counts, and HIV plasma viral load. In separate GEE regression analyses, V1V2 length was significantly correlated with time since infection (β = 1.00 increase in V1V2 length per year; p<0.001, Figure 2, upper panel) and clinical stage, as subjects with stage 3 (β = 6.36; p<0.001) and stage 4 (β = 3.30; p = 0.02), but not stage 2 (β = 0.80; p = 0.4) had significantly longer V1V2 lengths compared to subjects with stage 1 infection (Figure 3 and S12). However, V1V2 length did not significantly correlate with either CD4 stratum (<200, 200–500 or >500 cells/ml) or plasma viral load.

#### Relationship between V1V2 loop length, sample features and clinical factors – multivariate analyses (Table 3)

To further understand the interaction between significant variables, we next performed multivariate analyses of V1V2 length vs. time since infection, clinical stage, CD4 level, and HIV viral load after adjusting for calendar year and type of sample. This analysis was performed for all sequences in the dataset, as well as with plasma sequences and PBMC sequences considered separately (Table 3). Overall, V1V2 length was not significantly associated with time since infection, CD4 level, or HIV viral load. However, among sequences derived from plasma, V1V2 length was significantly associated with increased time since infection (β = 0.77 per year; p<0.001). Conversely, among the PBMC sequences, V1V2 length was associated with decreased CD4 counts (β = 8.13 for CD4 counts between 200 and 500 and β = 6.77 for CD4 counts less than 200 compared to >500) although the association with the lowest CD4 count group did not reach statistical significance (p = 0.09). Among subjects without AIDS (Stages 1 through 3), V1V2 length was associated with time since infection (β = 0.70 increase in V1V2 length per year; p<0.001), even after adjustment for calendar year and type of sample (data not shown). Overall, after adjusting for calendar year and sample type, V1V2 length remained significantly associated with clinical stage, as subjects with stage 3 (β = 6.25; p<0.001) and stage 4 (β = 3.54; p = 0.02), but not stage 2 (β = 0.09; p = 0.9) had significantly longer V1V2 lengths compared to subjects with stage 1 infection. However, V1V2 lengths in subjects with clinical stage 4 were significantly shorter than V1V2 lengths from subjects in stage 3 (p<0.001). The findings of increased V1V2 length in stage 3 and 4 infection compared to stage 1 and 2 were similarly noted both among sequences derived from plasma as well as PBMC, although the plasma associations did not reach statistical significance in all cases. In order to assess the potential that the results regarding clinical and viral factors associated with V1V2 length could be driven by unusually short or long sequences, we repeated the above analyses excluding the shortest and longest 5% of V1V2 lengths. Since model coefficients and p-values were similar in this restricted analysis (Table S2), our findings do not appear to be unduly influenced by a small number of outlying small or large sequences (Also see Text S1, section S3 and Figure S6).

**Table 3:** Multivariable regression analysis of V1V2 length vs. clinical variables.

	Time since infection (Model 1)			Infection Stage (Model 2)		
Factor	All seqs	Plasma	PBMC	All seqs	Plasma	PBMC
**Time since infection**	0.59 (0.85)	0.77 (<0.001)	0.56 (0.86)			
**Stage 1**				Ref	Ref	Ref
**Stage 2**				0.09 (0.9)	0.08 (0.9)	0.00 (0.9)
**Stage 3**				6.25 (<0.001)	6.65 (<0.001)	3.66 (0.10)
**Stage 4**				3.54 (0.02)	2.95 (0.06)	8.01 (0.01)

Factor	CD4 count (Model 3)			Viral Load (Model 4)		
CD4 level	All seqs	Plasma	PBMC	All seqs	Plasma	PBMC
**>500**	Ref	Ref	Ref			
**200-500**	0.87 (0.5)	0.45 (0.7)	8.13 (0.03)			
**<200**	−0.19 (0.9)	−0.70 (0.7)	6.77 (0.09)			
**VL log10 (continuous)**				−0.61 (0.14)	−0.58 (0.2)	−0.84 (0.15)

Beta coefficients for V1V2 Length vs. Time since Infection (Model 1), Stage of Infection (Model 2), CD4 counts (Model 3) or HIV Viral Load (Model 4). β values and p-values (in parentheses) are shown. Results are stratified by sample type (Plasma vs. PBMC), adjusting for year of sample collection. Time since infection was missing for 5 sequences, stage of infection for 242 sequences, CD4 count for 113 sequences, and viral load for 290 sequences with measured V1V2 length. Ref  =  Reference group. Analyses were performed for all sequences collectively as well as for sequences derived from plasma and PBMC considered separately.

As an alternative means of accounting for the variable number of sequences contributed by study subjects, the data was subjected to a resampling analysis, in which each subject contributed a single randomly selected sequence. This process was repeated 100 times, resulting in 100 resampled datasets. These analyses confirmed that the observed relationship between V1V2 length and time since infection, and year of sampling were not significantly biased due to the inclusion of individuals with multiple sequences (See Text S1, section S2 and Figure S5).

#### Relationship between V4 and V5 loop lengths and clinical variables

(Also see Text S1, section S4 and Figure S7.) Despite their high degree of length variability, V4 and V5 loop lengths did not appear to vary significantly by time since infection in univariate regression analyses. In separate analyses adjusting for sample year and type, V4 length appeared somewhat increased in those with stage 2 (β = 1.22, p = 0.02), stage 3 (β = 0.98, p = 0.09) or stage 4 (β = 1.11, p = 0.10) compared to stage 1 infection. In contrast, V5 length decreased with increasing time after infection (β = −0.07, p<0.001), was decreased in stage 4 (β = −0.69, p = 0.01 compared to stage 1), and was decreased in those with CD4 counts below 200 cells/ml (β = −0.66, p = 0.002) compared to those with CD4 counts above 500 cells/ml.

#### Relationship between subregion glycosylation and clinical variables

(And see Text S1 section S1, and Figures S3, S4 and S8.) In separate univariate GEE analyses, the number of PNLGS in V1V2 increased with calendar year of sampling (β = 0.06 increase per year; p = 0.02), but was not significantly associated with sample type (β = 0.32 more potential sites in PBMC compared to plasma; p = 0.17). Glycosylation in V1V2 was increased in those with stage 3 (β = 0.96, p = 0.002), but not stage 2 or 4 compared to stage 1 infection, and was decreased in those with CD4 counts <200 cells/ml (β = −0.63, p = 0.04 compared to CD4 counts >500). Similar findings were obtained in an analysis restricted to sequences derived from plasma; the number of PNLGS in V1V2 increased with calendar year of sampling (β = 0.05 increase per year; p = 0.001), was increased in those with stage 3 infection (β = 1.14, p = 0.001) and was decreased in those with CD4 counts <200 cells/ml (β = −0.84, p = 0.04 compared to CD4 counts >500). However, in PBMC, the number of sequences was limited, and no associations between the number of potential glycosylation sites and clinical features achieved statistical significance. Glycosylation in V4 decreased (p<0.001), while in V5 glycosylation increased with calendar year (β = 0.01 per year, p = 0.02), although the magnitude of these effects was small (β = −0.03 per year, and 0.01 per year, respectively).

#### Coreceptor usage, clinical factors and V1V2 loop length

(Also see Text S1 section S5 and Figures S10 and S11.) We next used four published genotypic methods to infer coreceptor usage based on V3 loop amino acid sequence [62]–[65]. In our dataset, 4476 V3 loop sequences were available for scoring, and were derived from 129 individuals. 121 V3 loops could not be scored by the PGRC method because the aligned sequences exceeded the length limit specified by the input format (40 characters). There was agreement in coreceptor usage assignment by all of the methods in 3644 of 4476 sequences (81.4%) and disagreement between one or more methods in the remaining 832 sequences. 1046 of 4476 sequences were scored as CXR4-using or syncytium-inducing by one or more methods, and the remaining 3430 were uniformly scored as CCR5 or non-syncytium by all methods. 60 of 129 individuals had at least one X4-scoring V3 loop as determined by one or more of the prediction methods, while the remaining 69 had only CCR5-scoring sequences.

We then considered inferred coreceptor usage as a function of time since infection, clinical stage, CD4 counts, HIV viral load, and V1V2 length, both overall and separately in plasma- and PBMC–derived viruses. Because the PSSM method provides a continuous numerical measure corresponding to the sequence position on a continuum of the evolutionary changes leading to X4 usage (the PSSM score), we examined PSSM score in relation to these variables. Overall, in separate GEE regression analyses, PSSM score was not related to time since infection (p = 0.9) or HIV viral load (p = 0.5). However, PSSM score was significantly increased (indicating greater CXCR4 usage) in those with stage 4 (β = 6.34, p = 0.0002) but not stage 2 or stage 3 infection (p = 0.8 each). Similarly, PSSM score was significantly increased in those with intermediate (200–500 cells/ml) and low (<200 cells/ml) CD4 counts (β = 1.52, p = 0.02 and β = 6.62; p<0.0001, respectively) compared to those with CD4 counts above 500 cells/ml. PSSM score was weakly associated with increased V1V2 length (β = 0.06; p = 0.09 per one amino acid increase in V1V2 length). The analyses restricted to plasma samples yielded similar results, with PSSM score strongly associated with stage 4 infection (β = 8.54, p<0.0001), intermediate (200–500 cells/ml) and low (<200 cells/ml) CD4 counts (β = 1.67, p = 0.03 and β = 8.83; p<0.0001, respectively) compared to those with CD4 counts above 500 cells/ml, and PSSM score weakly associated with increased V1V2 length (β = 0.07; p = 0.12 per one amino acid increase in V1V2 length). In sequences derived from PBMC samples, PSSM score was not associated with stage of infection, CD4 counts, HIV viral load, or V1V2 length. However, PSSM score was inversely associated with time since infection (β = −0.15; p = 0.01 per year).

### Longitudinal analyses

**Figure 4:**
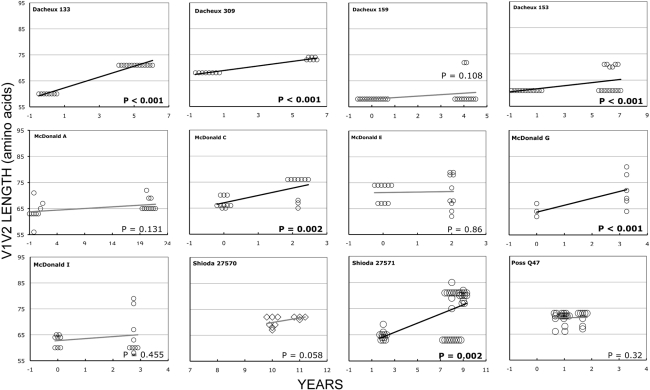
V1V2 loop lengths over time in group L1. Sequences from plasma are represented by diamonds and sequences from PBMC are represented by circles. Significant slopes are indicated in bold. X-axis denotes years elapsed between sampling time points, but do not necessarily indicate the total duration of infection. The first author of the report in which data were originally presented is indicated in the upper left-hand corner of each graph. Group L1 subjects did not meet criteria for AIDS at any time prior to the final sample. Subjects reported by McDonald et al had received AZT monotherapy at one or more times prior to sampling.

**Figure 5:**
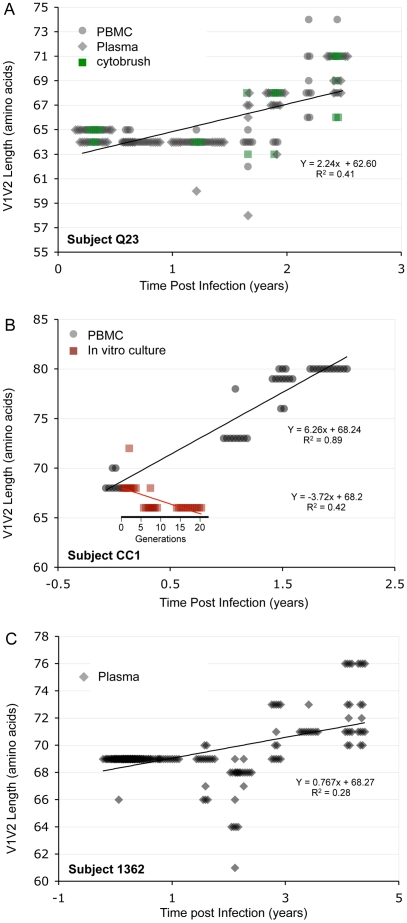
V1V2 length *vs*. time in subjects Q23, CC1 and 1362. Panel A: Subject Q23, infected with HIV subtype A. Sequences were derived from PBMC (black circles), plasma (black diamonds) and DNA from cervical lymphocytes (green squares) as described by Poss et al [80]. Panel B: Subject CC1, infected with subtype A. Sequences were obtained from plasma (black diamonds) and tissue culture (red squares). Length change of *in vitro* sequences occurs over ∼ 40 days, and are represented along an expanded X-axis for clarity.

**Figure 6:**
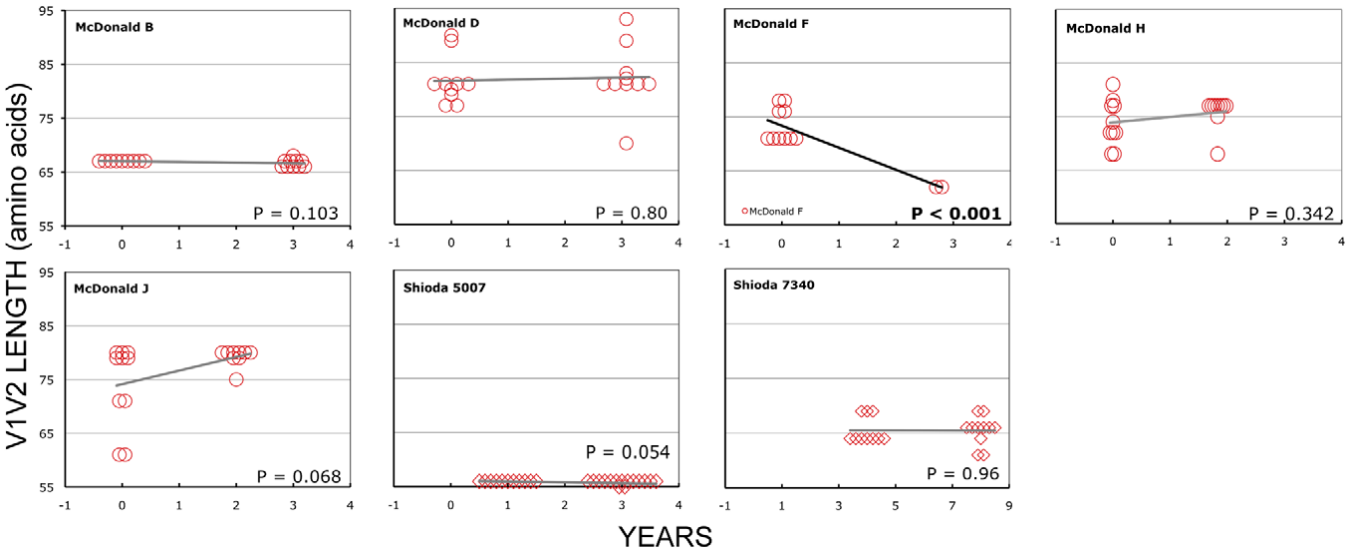
V1V2 loop lengths over time in group L2. Sequences from plasma are represented by diamonds and sequences from PBMC are represented by circles. Significant slopes are indicated in bold. X-axis denotes years elapsed between sampling time points, but do not necessarily indicate the total duration of infection. The first author of the report in which data were originally presented are indicated in the upper left-hand corner of each graph. Group L2 subjects were reported to have an AIDS-defining illness or peripheral CD4 count <200/mm^3^ between the first and second samples.

In the longitudinal dataset, significant V1V2 length increases between first and second timepoints were noted in 10 of 22 subjects, a significant V1V2 length decrease over time occurred in one subject, and no significant V1V2 length changes over time were seen in the remaining 11 subjects. These findings appeared to vary by stage of infection (t-test p = 0.03). In the 15 patients from the L1 group (individuals not meeting AIDS criteria at any time prior to final sampling), the mean increase of V1V2 length per subjects was 1.69 amino acids per year, and 9 subjects experienced significant V1V2 length increases over time (Figures 4 and 5). In contrast, of the seven subjects in the L2 group (individuals progressing to AIDS between first and final sample), the mean V1V2 length decreased by an average of 0.10 amino acids per year, with only one having a significant trend of increasing length, while one individual showed a significant decrease in length (Figure 6). The distribution of V1V2 length change (increase or decrease) by group was therefore asymmetric (Fisher's exact test, p = 0.02), reflecting a trend of increasing length in asymptomatic individuals (group L1) and stable or decreasing length in individuals with AIDS (group L2) (Table 4). Three subjects in group L1 had extensive longitudinal sampling (Figure 5); in 1362 and Q23 [51], there was a period of V1V2 length stability of approximately 2 years, followed by increase through 4.5 years. V1V2 length increase over time was also seen in CC1. In the case of CC1, a pseudotyped virus was created using the gp120 coding region from the initial timepoint from this individual in a HIV-1 NL4-3 background, and cultured *in vitro* [54]. In contrast to the patterns observed *in vivo*, V1V2 length and number of glycosylation sites both declined rapidly over 20 generations *in vitro* (p<0.001).

**Table 4:** Summary of longitudinal data.

ID	Slope	p-value	Group
133	2.1774	**<.0001**	L1
1362	0.7478	**<.0001**	L1
153	0.6097	**0.0016**	L1
159	0.5801	0.0943	L1
27570	1.8929	0.0566	L1
27571	1.8123	**<.0001**	L1
309	0.8926	**<.0001**	L1
A	1.25	0.0575	L1
C	2.8704	**<.0001**	L1
CC1	6.29	**<.0001**	L1
E	0.2	0.8591	L1
G	2.6462	**<.0001**	L1
I	0.7778	0.4324	L1
Q23	2.4755	**<.0001**	L1
Q47	0.1087	0.808	L1
5007	−0.1765	0.0856	L2
7340	−0.0114	0.9655	L2
B	−0.1333	0.0704	L2
D	0.1948	0.788	L2
F	−4.1455	**<.0001**	L2
H	1.0261	0.2487	L2
J	2.5611	**0.0401**	L2

Initial parameter estimates and p-values were used when only two time points were available. When multiple timepoints were available, final GEE estimates and p-values were used. P-values less than 0.05 are shown in bold.

## Discussion

We have systematically examined gp120 subregion length variation, and the relationship between length polymorphism, N-linked glycosylation sites, and clinical markers of disease progression. Although V1V2, V4 and V5 all displayed remarkable length heterogeneity, and V1V2, C3 and V4 were also quite variable with respect to glycosylation, the most significant associations between virological and clinical variables localized to the V1V2 region. We found that V1V2 length and glycosylation increased significantly over time during chronic infection, and then declined in late-stage illness. In regression analyses, time since infection was the most influential factor in determining V1V2 length. In addition, there was a modest but significant increase in V1V2 length over the period from 1984–2004. V5 loop length was highly variable, but tended to decrease slightly in length over the course of infection.

In SIV infection, the number of PNLGS in gp120 increases over time *in vivo* following inoculation of a cell-passaged strain [71]. In one earlier study in humans, Bunnik et al noted expansion in gp120 length followed by contraction over time in 4 of 5 individuals receiving antiretroviral therapy, and similar changes in glycosylation in 3 subjects [72]. Others have noted a relationship between early infection and reduced V1V2 length and glycosylation in subtypes C and A [19], [45]. In contrast, a comparison of early and chronic HIV-1 subtype B sequences from the HIV sequence database failed to reveal any significant difference in V1V2 length [19], suggesting that these effects may be subtype-specific. Data on length/glycosylation changes during transmission have been conflicting. Derdeyn et al [45] demonstrated reduced length and glycosylation in V1–V4 following heterosexual transmission in HIV-1 subtype C. However, Frost et al failed to note similar findings in a study of eight subtype B homosexual transmission pairs [47], and in our examination of these and 10 additional subtype B infected homosexual transmission pairs, we found no consistent pattern of change in V1–V2 or V1–V4 length or glycosylation upon transmission [46].

Interpretation of the data presented here may be affected by several methodological factors. There is probably some variation in the accuracy of the reported time of infection for sequences obtained from previous reports. In some cases, sequences obtained from prior publications may have been obtained under conditions permitting template resampling [73], and a systematic error due to evolving laboratory methods could result in bias. Also, in our analyses, we have not formally corrected for multiple comparisons. Physiological factors are also likely to introduce some noise, particularly in cross-sectional analyses of parameters with respect to time since infection. The individuals included here represent a broad spectrum of clinical scenarios, diverse host immune response profiles and varying disease progression rates. Plasma sequences may receive contributions from both recently infected target cells and older reservoirs, and therefore imperfectly reflect selective pressures prevailing at the time of infection. Finally, length and glycosylation phenotypes are likely to be affected by chance events and unknown factors not considered in our analyses. Therefore, the effects we describe are influential rather than deterministic, and reflect important selective forces that can be discerned against a background of high inter-individual variation.

**Figure 7:**
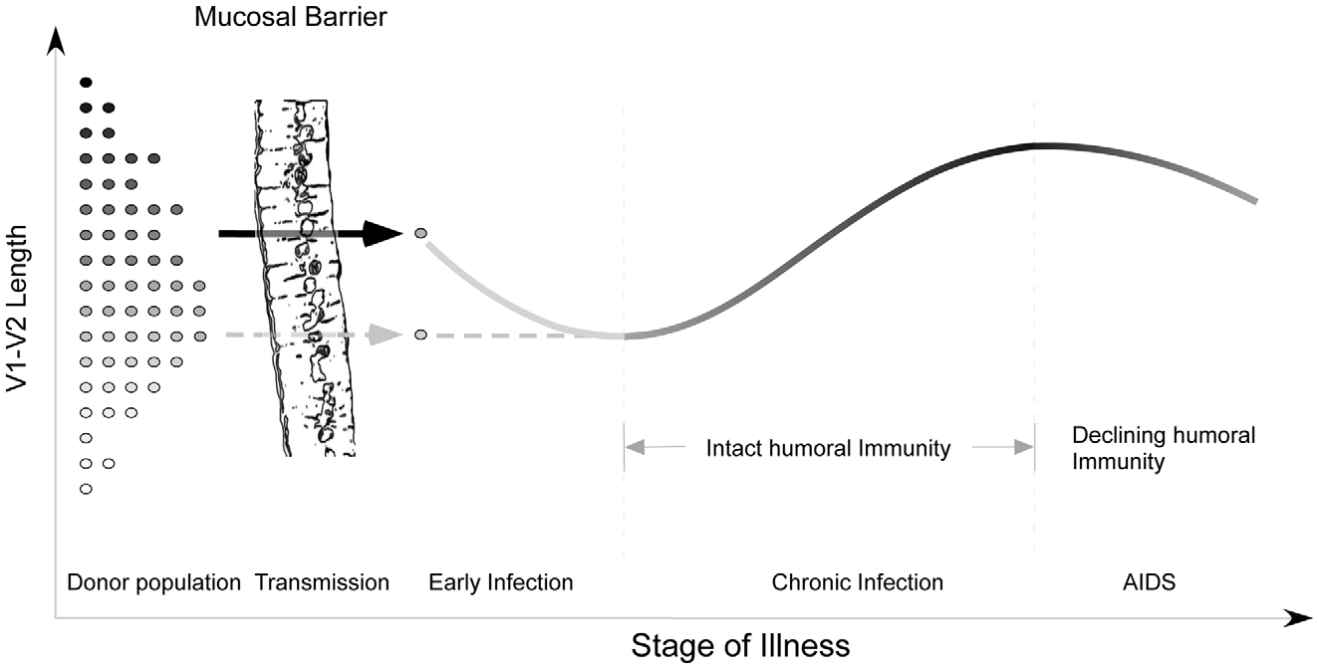
Proposed evolution of V1V2 loop size change during transmission and HIV infection. At the time of sexual transmission, a significant genetic bottleneck occurs in which one or a small number of donor variants is transmitted to the recipient, without clear selection for loop size (represented on the y-axis). During early infection, prior to an effective host response, viral variants with a compact V1V2 loop have a competitive advantage, and V1V2 loop size remains stable or regresses. During chronic asymptomatic infection, mean V1V2 length increases in response to (humoral) immune selective pressure. As immune function wanes, V1V2 loop length gradually declines.

Despite these limitations, the analyses presented here and the work of others [40], [45]–[47], [72] provide the outlines of an overall pattern characterized by transmission of randomly selected V1V2 loop lengths from viruses present in the donor pool, a brief decline in loop size during the initial months immediately following infection, gradual selection for bulkier V1V2 loops during chronic infection, and finally, reversion to more compact loops during late stage illness. Structural studies [22], [23], neutralization studies [20], [34]–[38], [42], and *in vitro* data on viruses lacking V1 and V2 [43], [44] suggest that one major function of the V1V2 region may be to permit evasion from humoral immune responses in the host. Thus, the trends outlined above support the hypothesis that HIV populations may evolve to escape humoral selective pressure by increasing V1V2 loop size. According to this view, the newly infected, immunologically naïve host might be expected to harbor relatively short V1V2 loops that eventually lengthen in response to an effective humoral response at some fitness cost (Figure S9). Experimental evidence indicating that relaxation of antibody-mediated selective pressure during early infection is associated with shorter loops is provided by Derdeyn, who demonstrated significantly greater neutralization sensitivity among five recipients during early infection, than in the corresponding donors [45]. The decline in V1V2 size observed in advanced disease probably reflects waning effectiveness of humoral immunity in hosts with late-stage illness and profound immune dysregulation (Figure 7). This decline is also congruent with previous findings of an inverse relationship between the rate of HIV genetic evolution and the rate of CD4 T cell decline in some individuals [74]. The dramatic reduction in V1V2 length associated with transfer to the *in vitro* environment [54] represents the extreme case of absent host immunity, where viruses without an unnecessarily bulky V1V2 loop achieve maximum replicative fitness. As would be expected, the patterns we observe are most pronounced in plasma sequences, which most directly reflect the selective forces present at the time of sampling. In contrast, a significant increase in V1V2 length over time was not seen in the PBMC compartment. These observations are consistent with the presence of archived genotypes from earlier times during the course of infection within the PBMC compartment. We also note that genotypes present in plasma may emanate from other cellular compartments in addition to PBMC, and may therefore reflect somewhat different evolutionary pressures. However, a considerably greater number of V1V2 sequences were derived from plasma, and sample size may also account for some of the differences observed between these compartments.

Our model may help to explain a failure to find any significant difference in V1V2 length in a comparison of early and chronic HIV-1 subtype B sequences (including sequences from late-stage individuals) [19]. When we reanalyzed the data presented by Chohan [19] after separating subjects with stable chronic illness from subjects with AIDS (Figure S13), we observed a pattern of lengthening over time, followed by decline in late-stage illness, as reported here (See Text S1, section S7). Similarly, we may explain discordant results obtained on V1V2 length variation during transmission of HIV-1 subtypes C and B. While a trend towards shorter loops in recipients was seen in subtype C [45] but not B [46], [47], it is likely for methodological reasons that the subjects studied by Derdeyn were sampled at somewhat later times than those of Frost and Liu. Thus the sequences in the latter two studies would be expected to be a random sampling from the donor pool, while those of Derdeyn might reflect the expected shortening prior to the onset of an effective antibody response. Indeed, when we examine a much larger set of subtype A and C transmission pairs from East Africa with more precisely known sampling times obtained soon after transmission, it is difficult to appreciate any consistent pattern of V1V2 length change (See Text S1, section S8 and Figure S14). Thus there may be no need to infer separate mechanisms for different HIV-1 subtypes and modes of transmission.

In addition, we may also explain a trend of increasing V1V2 length by calendar year. If shorter and less glycosylated V1V2 were always selected during transmission, transmission from donors in early infection would maintain a constant V1V2 length within the epidemic, whereas if all new cases were acquired from chronically infected hosts, this increase of V1V2 length by calendar year could be dramatic. However, most studies suggest that about half of transmission events involve subjects in early infection [46], [75], [76], consistent with the moderate trend we observed. Alternatively, the temporal trends we have observed could represent a gradual adaptation by HIV-1 to host the host environment at the population level, a hypothesis that has been proposed by several investigators with respect to mutational escape from HLA-restricted CTL epitopes [77]–[79].

Finally, our results imply that the polymorphisms seen in V1V2 reflect the ability of the host to mount a meaningful immunological response, rather than virologic features that dictate the course of illness. That is, we argue that V1V2 length change is a consequence of environmental selective pressure rather than a causative factor in disease progression.

## Supporting Information

Text S1 Supporting analyses text - PDF document containing text containing supplementary analyses and citations.
(0.14 MB PDF)

Figure S1 V1V2 length vs. virologic and clinical parameters I. **Panel A:** V1V2 length vs. log_10_ plasma viral load (no significant relationship). **Panel B:** V1V2 length vs. peripheral CD4 T-cell count (no significant relationship). **Panel C:** V1V2 length by coreceptor usage. Box-plots depict minimum, 1^st^ quartile, median (red line), 3^rd^ quartile and maximum values in each group, with superimposed individual length measurements. In this series, V1V2 sequences associated with V3 loops predicted to be X4-tropic by PSSM are slightly longer compared with sequences associated with R5-tropic V3 loops (median 71 vs. 66 amino acids, p = 3.49×10^-5^, MW test). However, a plot of V1V2 length vs. PSSM score (**Panel D**) does not reveal a clear linear correlation between V1V2 length and PSSM score.
(1.15 MB TIF)

Figure S2 V1V2 length vs. virologic and clinical parameters II. **Panel A:** V1V2 length vs. time since infection. As described earlier, a significant positive correlation V1V2 length and time since infection is evident (R = 0.149) **Panel B:** V1V2 length by stage of infection. Box-plots depict minimum, 1^st^ quartile, median (red line), 3^rd^ quartile and maximum values in each stage group, with superimposed individual length measurements. Highly significant differences in V1V2 length are seen between stage 3 and stages 1,2 and 4 (p<2.2×10^−16^, M-W rank sum test), reflecting V1V2 lengthening in chronic illness, followed by contraction in late disease. **Panel C:** V1V2 length by site (PBMC vs. plasma). In this univariate comparison, there is no significant length difference between V1V2 loops obtained from PBMC (median 68 amino acids) and plasma (median 66 amino acids, p = 0.93). **Panel D:** V1V2 length vs. year of sampling. As described, there is a significant positive correlation between V1V2 length and year of sampling.
(1.14 MB TIF)

Figure S3 V1V2 glycosylation vs. virological and clinical parameters I. **Panel A:** Number of V1V2 glycosylation sites vs. log_10_ plasma viral load (no significant relationship). **Panel B:** Number of V1V2 glycosylation sites vs. peripheral CD4 T-cell count (no clear correlation observed). **Panel C:** V1V2 glycosylation sites by inferred coreceptor usage (R5 or X4). Box-plots report minimum, 1^st^ quartile, median (red line), 3^rd^ quartile and maximum values in each stage group, with superimposed individual measurements. No clear differences in glycosylation are noted between V1V2 loops associated with R5-tropic and X4-tropic V3 loops (median 6 and 6 PNLGS, respectively). **Panel D:** Number of V1V2 glycosylation sites vs. PSSM score (no significant relationship).
(1.07 MB TIF)

Figure S4 V1V2 glycosylation vs. virological and clinical parameters II. **Panel A:** Number of V1V2 glycosylation sites vs. time since infection. As with V1V2 length, in a univariate analysis there is a modest but significant linear correlation between time since infection and the extent of V1V2 glycosylation (β = 0.12 amino acids/year, R^2^ = 0.09). **Panel B:** Number of V1V2 glycosylation sites by clinical stage. Similar to what was observed for V1V2 length, glycosylation in chronic illness (stage 3) was significantly greater than in early and late disease (p<1×10^−8^), reflecting increasing glycosylation during chronic infection, followed by a decline in the extent of glycosylation during AIDS. **Panel C:** V1V2 glycosylation sites by site (PBMC or plasma). Box-plots report minimum, 1^st^ quartile, median (red line), 3^rd^ quartile and maximum values in each stage group, with superimposed individual measurements. No clear differences in glycosylation are noted between V1V2 loops obtained from PBMC vs. plasma (median PNLGs 5 and 6, respectively, p = 0.59). **Panel D:** Number of V1V2 glycosylation sites vs. year of sampling. There is a negligible positive correlation between V1V2 PNLG and year of sampling (β = 0.05, R^2^ = 0.06)
(1.13 MB TIF)

Figure S5 Resampling analysis: R^2^ values for multiple linear regression of V1V2 length on the independent variables time since infection, year of sampling, and sample type for the entire dataset (red squares □) and for 100 parallel randomly resampled datasets derived from the original dataset (green diamonds ⋄). Correlation coefficients obtained in the resampled datasets were consistent with the correlation coefficient obtained using all data.
(1.33 MB TIF)

Figure S6 V1V2 sequence length vs. time since infection - sliding window analysis: Length measurements (red + sign) and R^2^ values (blue triangles Δ) for univariate linear regression analyses of datasets excluding 0.4-year periods since the time of infection. 0.4-year data exclusion periods are centered around the x value of each Δ datapoint. The correlation strength of the linear model is greatest for datasets excluding the earliest two 0.4-year periods (first two datapoints), indicating that linear regression of V1V2 length on time since infection most accurately explains data obtained at times after approximately 0.8 years.
(1.07 MB TIF)

Figure S7 Subregion length (V1–V5) vs. time since infection for the V1V5 region (purple circles), the V1V4 region (green diamonds) and V1V2 (blue squares), V4 (red triangles) and V5 (orange diamonds) considered separately. A significant trend towards increasing length seen in V1V2, V1V4 and V1V5 can be ascribed primarily to changes in V1V2.
(2.13 MB TIF)

Figure S8 Subregion glycosylation (V1–V5) vs. time since infection for the V1V5 region (purple circles), the V1V4 region (green diamonds) and for V1V2 (blue squares) and V4 (red triangles) considered separately. A modest trend towards increasing glycosylaton seen in V1V2, V1V4 and V1V5 can be ascribed primarily to changes in V1V2
(2.38 MB TIF)

Figure S9 Agreement between 4 bioinformatic coreceptors used to assign probable coreceptor usage. There was complete agreement between all methods for ∼80% of sequences examined, while in the remaining 20%, there was some disagreement in assignment between one or more scoring methods. Most sequences were predicted to be CCR5-tropic by all methods (white bar), while a modest number of sequences was predicted to be CXCR4-tropic by all methods. The remaining sequences were scored differently by various methods, as represented (colored bars).
(2.84 MB TIF)

Figure S10 V1V2 sequence length *vs.* time since infection and PSSM score. Rising PSSM scores (color scale), depicted as warmer colors, indicate a greater likelihood of CXCR4 coreceptor usage; in this dataset, predicted X4 coreceptor usage occurs at a PSSM score of approximately -2. In these data, there is a pronounced preponderance of CCR5-using viruses, with a trend towards increasing prevalence of X4-tropic viruses during chronic infection. However, X4 and R5 viruses are distributed throughout all infection times, and cannot be easily distinguished on the basis of V1V2 length.
(1.15 MB TIF)

Figure S11 V1V2 potential N-linked glycosylation sites *vs.* V1V2 length and PSSM score (color scale). There is a very marked dependence of glycosylation on length (β = 0.13 PNGL/amino acid, R^2^ = 0.52). X4-usage appears to be more commonly associated with V1V2 sequences bearing 4-7 PNLG sites, than with sequences with more than 7 sites (and see figure S1 panel D).
(1.10 MB TIF)

Figure S12 V1V2 and Stage of Illness. V1V2 length vs. Time since Infection for stage 1 (orange “+”), stage 2 (gray triangles), stage 3 (blue squares), and stage 4 (red diamonds). There is a slight decline in V1V2 length from stage 1 to stage 2, reflecting regression from transmitted viruses of essentially random lengths to shorter loop lengths during early infection prior to the onset of a meaningful immune response. This is followed by a strong trend towards lengthening during chronic infection (stage 3) and a weakening of this trend in late-stage illness (stage 4).
(1.52 MB TIF)

Figure S13 Chohan Data revisited: V1V2 sequence length for subjects in early infection (first bar), chronic infection and AIDS considered together (second bar), chronic stable infection only (third bar), and individuals with AIDS-defining clinical conditions (fourth bar). Length differences between “early”, “chronic” and “AIDS” are statistically significant (p≤0.02). Thus, separation of sequences obtained during AIDS from sequences obtained during chronic stable infection reveals a trend of rising V1V2 length through chronic infection, followed by falling length in AIDS that is not otherwise apparent.
(1.04 MB TIF)

Figure S14 V1V2 length during transmission: Change in mean loop length between donor and recipient in 44 transmission pairs involving HIV-1 subtypes A, C and B, presented by Haaland, Derdeyn, Frost and Liu. **Panels A–C**: Difference in mean loop length between donors and recipients vs. time since infection for V1V2 (**panel A**), C2–V4 (**panel B**), and V1–V4 (**panel C**). **Panel D**: Difference in mean loop length between donors and recipients vs. the mean loop length (for the corresponding region) in the donor. Subtype A sequences (Haaland, represented by red +), Subtype B sequences (Frost, blue X) and Liu (blue squares) and subtype C sequences (Haaland, green squares, and Derdeyn, green X).
(2.25 MB TIF)

Table S1 Published sequence data. Accession Numbers for previously published sequences included in cross-sectional, longitudinal and transmission analyses.
(0.05 MB PDF)

Table S2 Multivariable regression analysis of V1V2 length vs. clinical variables, upper and lower 5% excluded. Beta coefficients for V1V2 Length vs. Time since Infection (Model 1), Stage of Infection (Model 2), CD4 counts (Model 3) or HIV Viral Load (Model 4). β values and p-values (in parentheses) are shown. Results are stratified by sample type (Plasma vs. PBMC), adjusting for year of sample collection. Time since infection was missing for 5 sequences, stage of infection for 242 sequences, CD4 count for 113 sequences, and viral load for 290 sequences with measured V1V2 length. Ref = Reference group. Analyses were performed for all sequences collectively as well as for sequences derived from plasma and PBMC considered separately. Sequences comprising the upper and lower 5% by length were excluded from these analyses.
(0.06 MB PDF)
