# Supplementary material for: HIV-1 Envelope Subregion Length Variation during Disease Progression
Source: PLoS Pathog. 2010 Dec 16;6(12):e1001228. doi: 10.1371/journal.ppat.1001228 (PMC3002983; doi:10.1371/journal.ppat.1001228)
Supplement: Text S1 — Supporting analyses text - PDF document containing text containing supplementary analyses and citations. (0.14 MB PDF) [file ppat.1001228.s001.pdf]

SUPPLEMENTARY ANALYSES

In the following sections, we provide additional graphical representations of our data and analyses intended to allow for a more intuitive understanding of the relationships between the gene sequence data and clinical information presented in our manuscript. We first present plots of V1V2 length and glycosylation as they relate to the other virological and clinical parameters examined in this work (Section 1). In later sections we explore in greater detail the relationships between V1V2 features and the significantly related parameters ***time since infection***, ***sample type*** (i.e. plasma or PBMC), ***year of sampling*** and the virological parameters ***V1V2 length***, ***V1V2 glycosylation sites*** (i.e. the number of potential N-linked glycosylation sites within the V1V2 sequence) and ***PSSM score*** (i.e. the score assigned to the associated V3 loop sequence by the position-specific scoring matrix), which estimates the likelihood of CCR5 and CXCR4 coreceptor tropism [1].

In cases where we wished to isolate the effect of individual clinical variables, we performed simple linear regression with a single variable, and reported the proportion of the variability in the data that is accounted for by this variable (i.e., R^2, the coefficient of determination) across selected ranges of the entire dataset. For these analyses, we utilized the entire V1V2 dataset comprised of N = 1690 sequences (plasma N = 1537, PBMC N = 153) wherever possible. An associated V3 loop sequence (and therefore PSSM information) was available for 1391 of 1690 sequences (plasma N = 1331, PBMCN = 60). Year of sampling was available for 1666 of 1690 sequences (plasma = 1331, PBMC = 36). Contemporaneous plasma viral load and peripheral CD4+ T-cell count information was available for 1638 and 1624 sequences, respectively. Analyses involving these parameters were performed using the corresponding data subsets. Linear regression was performed using the R statistical software package.

1. GRAPHICAL REPRESENTATION OF DATA

We first wished to provide additional graphical representation for the relationships between V1V2 length and the clinical parameters ***viral load***, ***CD4 count***, ***PSSM score*** and ***predicted coreceptor usage*** **(**Figure S1**)**. No significant relationships were evident between V1V2 length and either plasma viral load or peripheral CD4+ T-cell count (R^2 values and correlation coefficients approximating 0) (Figure S1, panels A and B). There was a significant trend towards shorter V1V2 loops in sequences associated with R5-tropic vs. X4-tropic V3 loops (median 66 vs. 71 amino acids, $p = 3.49 \times 10^{-5}$ Mann-Whitney rank sum test)(Figure S1, panel C). This raised the possibility that there might be a negative correlation between V1V2 length and PSSM score. However, a univariate model of length on PSSM score did not yield a clear relationship. PSSM score and coreceptor usage were further explored in **Section 4**.

We next wished to graphically represent V1V2 vs. the parameters ***time since infection***, ***stage***, ***site*** (i.e. PBMC vs. plasma) and ***year of sampling*** (Figure S2). As described previously, there was a significant positive correlation between ***V1V2 length*** and ***time since infection*** (β = 0.79 amino acids/year, R^2 = 0.15) (Figure S2, panel A). Length was also significantly associated with ***stage of infection*** **(**Figure S2, panel B). Individual length measurements were compared between stages 1-4 using the Mann-Whitney rank sum test, revealing highly significant differences between stage 3 (longer V1V2 loop length) and stages 1,2 and 4, reflecting a rise in loop length during chronic illness, followed by V1V2 contraction in late-stage illness. There were no significant differences between **V1V2 length** by ***site*** (PBMC and plasma median lengths 68 and 66 amino acids, respectively, p = 0.96 Mann-Whitney rank sum test)(Figure S2, panel C). However a significant increase in ***V1V2 length*** is seen when regression is performed on ***year of sampling*** (β = 0.40 amino acids/year, R^2 = 0.15)**(**Figure S2, panel D). This effect remains significant in GEE analyses taking into account other significant variables. Possible explanations include sampling bias, and a true epidemiological trend towards greater lengths over time within the epidemic at large.

Because chain length and glycosylation may both contribute to V1V2 size, and could therefore plausibly respond in similar ways to common evolutionary pressures, we next performed analogous plots for V1V2 glycosylation vs. the parameters listed above (Figure S3). As with V1V2 loop length, we failed to observe any clear relationships between the number of V1V2 potential n-linked glycosylation sites (PNLGS) and ***viral load***, ***CD4 count***, ***PSSM score*** (Figure S3, panels A, B, D) or ***site*** (median V1V2 PNLGS for PBMC and plasma = 5 and 6, respectively, p = 0.59, Mann-Whitney Rank sum test)(Figure S3, panel C), but positive correlations with ***time since infection*** and ***stage*** (Figure S4, panels A and B)**. I**n contrast with the results for V1V2 length, there was only a negligible correlation between ***V1V2 glycosylation*** and ***year of sampling*** (β = 0.05, R^2 = 0.06) (Figure S4, panel D). The results presented in this section are consistent with the corresponding GEE analyses presented in our manuscript.

2. RANDOM RESAMPLING ANALYSIS

We next wished to provide an alternative means of determining whether the relationship between V1V2 loop properties and significantly correlated variables could be excessively influenced by a lack of independence between some sequences in the dataset. That is, could the associations we have observed be simply due to the overrepresentation of individuals contributing more than one sequence? To address this question, we first re-explored the V1V2 dataset by randomly selecting a single sequence from each individual, and repeating this 100 times to create 100 parallel data subsets derived from the original dataset. Both simple linear regression of ***V1V2 length*** vs ***time since infection*** and multiple linear regression of ***V1V2 length*** vs ***time since infection***, ***year of sampling*** and ***sample type*** were performed for each data subset.

We next repeated the resampling process by randomly selecting with replacement N1 individual measurements from each individual. As above, this was performed N2 times to create N2 parallel data subsets. For example, using N1 = 3 and N2 = 2, original data of the format;

SubjectA	Length1	-	-	-
SubjectB	Length1	Length2	Length3	Length4
SubjectC	Length1	Length2	-	-

becomes;

SubjectA	Length1	Length1	Length1
SubjectB	Length2	Length4	Length1
SubjectC	Length2	Length2	Length1

SubjectA	Length1	Length1	Length1
SubjectB	Length3	Length2	Length3
SubjectC	Length1	Length2	Length1

This approach permitted the use of all available sequence data while ensuring that there was no bias resulting from the inclusion of individuals with multiple sequences. Simple linear regression of ***V1V2 length*** vs. ***time since infection*** was performed for each resampled data subset. Resampling was performed for N1 values ranging from 1 – 10 and N2 values ranging from 10 to 1000 using a Perl script (available from the authors upon request).

*Results***:** For the 100 data subsets using N1 = 1, all available V1V2 data were included (N = 1690), and each resulting subset included 156 individual length measurements. In the univariate model of ***V1V2 length*** vs. ***time since infection***, the mean R^2 value obtained was 0.18 (range 0.11 to 0.27), consistent with the value of 0.16 in the original dataset. In the multivariate model, the mean R^2 values in the resampled dataset and the value obtained using the entire dataset were 0.30 and 0.28 respectively (Figure S5). In resampled data subsets using N1 > 1, significant correlations consistent with regression on the full dataset were also seen (data not shown).

Because V1V2 glycosylation was also noted to be correlated with the clinical parameters of interest in our GEE analyses, we next performed identical resampling analyses using univariate and multivariate linear regression relating the number of ***V1V2 glycosylation sites*** to ***time since infection***, ***sample year*** and ***sample type***, as above, using N1 = 1 and N2 = 100. As for V1V2 length, statistically significant correlations were noted in these models with closely matching R^2 values in the full data set and the 100 data subsets (data not shown).

Conclusions: Thus, eliminating data linkage by repeating our analyses using a large number of randomly resampled data subsets in which potentially nonindependent measurements were excluded yielded results entirely consistent with results derived from the entire dataset. From this we concluded that the covariances between length, glycosylation and time since infection that we observed were not an artifact of non-independence within the dataset.

3. LINEAR REGRESSION OF V1V2 LENGTH ON TIME SINCE INFECTION

We next wished to further dissect the relationship between time since infection and V1V2 length, to see if particular time ranges were more or less consistent with the linear models. We performed univariate linear regression of ***V1V2 length*** on ***time since infection*** while excluding sequence data collected within a sliding 0.4-year time window, ranging from 0 to 20 years. The goodness-of-fit for the optimal linear model relating ***length*** and ***time since infection*** for each restricted dataset was assessed by comparing R^2 values. This analysis was performed on V1V2 sequences derived from PBMC or plasma (N = 1690), and on “PBMC-only” (N = 153) and “plasma-only” (N = 1537) data subsets.

Results: R^2 values obtained from regression on each window-exclusion dataset were plotted (Figure S6, blue line). The coefficient of determination remained essentially constant for these datasets, with the exception of datasets in which sequences obtained from the first 0.8 years since infection were excluded. Omission of sequence length data from these early times resulted in a markedly improved fit of the univariate model. This pattern was obtained for both the “PBMC or plasma” and “plasma-only” datasets, but not for the “PBMC-only” dataset, where a majority of sequences (80/153) were obtained at times before 0.4 years post infection.

In view of these results concerning length and time since infection and our GEE analyses showing an unexpected dependence of V1V2 length on year of sampling, we performed similar exclusion-window analyses with a univariate linear model relating ***V1V2 length*** to ***year of sampling***. As in the case of ***length*** vs ***time since infection***, the best fit was obtained when data from the first 0.4 years following infection were excluded from the analysis (data not shown).

Conclusions: We see from these results that the linear regression models relating changes in the V1V2 region to clinical variables most accurately represent sequences obtained at times *beyond* early infection. In contrast, sequences obtained immediately after transmission appear to be highly variable and correlate poorly with measured clinical variables. This is consistent with an interpretation of early sequences reflecting highly disparate length variants “randomly” transmitted at the time of infection from donors, who themselves are likely to be at various stages of illness. As in our previous study, these data do not support a strong selective filter for long or short V1V2 length polymorphisms or particular glycosylation phenotypes at the time of transmission. However, over

time following infection, V1V2 features appear to trend towards length and glycosylation configurations that are optimal solutions for the selective conditions within the host.

4. THE ROLE OF SUBREGIONS OUTSIDE OF V1V2

Previous works addressing *env* changes during HIV transmission and disease progression have focused on the region spanning V1 through V4 rather than V1V2 [2,3,4]. We therefore wished to explore the relationship between envelope length/glycosylation and time since infection in *env* subregions downstream of V1V2, including C2, V3, C3, V4, C4 and V5. We reasoned that if these regions contribute to immune escape through loop length increase or by the addition of PNLG sites, a relationship between these variables and time should be evident in these regions individually. We therefore performed simple linear regression of ***length*** and ***glycosylation*** vs ***time since infection*** for C2 (N = 1270 sequences), V3 (N = 4402 sequences), C3 (n = 3611 sequences), V4 (N = 4403 sequences), C4 (N = 4400 sequences) and V5 (N = 4402 sequences) as well as for V1-V4 (N = 1225 sequences) and V1-V5 (N = 1225 sequences).

Results: We found that length change over time correlated most strongly with V1V2, but there was essentially no correlation with the remaining individual subregions. As expected, when downstream sequence information is added to V1V2, a correlation between time and length of V1V4 and V1V5 persists, but with weaker R^2 value, and these can be ascribed to changes in V1V2. (Figure S7). Similar results were obtained for glycosylation (Figure S8).

Region	β (length)	R^2 (length)	β (PNLG)	R^2 (PNLG)
V1V2	0.79	0.15	0.12	0.09
V1-V4	0.80	0.01	0.14	0.05
V1-V5	0.82	0.09	0.14	0.04
C2	0.00	0.00	0.00	0.00
V3	0.02	0.03	0.00	0.00
C3	0.04	0.03	0.00	0.00
V4	0.12	0.02	0.00	0.00
C4	0.00	0.00	0.00	0.00
V5	-0.04	0.02	0.00	0.00

Conclusions: We conclude from these analyses that loop size adaptations within gp120 over time during infection seem to occur primarily within V1V2, and that while other regions show some variability in length (particularly V4 and V5) and glycosylation (e.g., C2, V5), there are no consistent patterns of increase or decrease in these regions during disease progression. Adaptive changes within *env* regions downstream of V1V2, when they occur, might therefore involve changes in sequence identity and location of PNGL sites, rather than their absolute number.

5. CORECEPTOR USAGE

We next wished to further explore the relationships between clinical variables, V1V2 loop properties and inferred coreceptor usage of the associated V3 loop, as determined by four publicly available genotypic methods of predicting coreceptor usage [1,5,6,7], including PGRC, PSSM, Geno2Pheno (G2P), and BMLC respectively. We used the support vector machine user option for PGRC, and a standard false-positive cut-off of 10% for G2P. As noted elsewhere in the text, there was ~80% agreement between these methods (Figure 9).

Because the PSSM method also provides a numerical output corresponding to the progression from CCR5 to CXCR4 status, we next pursued the relationship between PSSM score and other genotypic parameters. We thought that both length and glycosylation changes could influence coreceptor usage, and therefore plotted ***V1V2 length*** vs. ***time since infection*** and ***PSSM score*** (Figure S10) and ***V1V2 glycosylation sites*** vs. ***V1V2 length*** and ***PSSM score*** (Figure S11**)**.

Results: In our dataset, PSSM scores greater than -1.97 were uniformly associated with a prediction of CXCR4 tropism, while values below this are nearly always predicted to be CCR5-tropic. 31 sequences were associated with X4 tropism, and an additional 7 sequences were associated with PSSM values ranging from -2 to -2.7, suggesting possible “transitional” sequences. These 38 sequences were all obtained from plasma. As expected, in a plot of ***V1V2 length*** vs. ***time since infection*** and ***PSSM score***, we saw a trend towards increasing prevalence of X4-troic viruses over time since infection, as has been previously described [8]. However, a small number of X4-tropic viruses were present in early infection. X4-tropic viruses were noted across nearly the full range of V1V2 length values, and only a weak relationship between ***PSSM score*** and ***V1V2 length*** was evident (Figure S10**)**. There was a strong linear correlation between ***V1V2 length*** and ***V1V2 glycosylation sites*** (Figure S11**)**. X4-tropic viruses were seen at glycosylation site numbers ranging from 4 to 7, but not observed in association with more heavily glycosylated sequences, aside from one sequence with 11 PNLG sites and a relatively low PSSM score (-4.13) that was nevertheless scored as X4-tropic.

Conclusions: There appears to be close linkage between V1V2 length and the number of potential N-linked glycosylation sites. X4-tropism appears to be infrequent in the context of highly glycosylated V1V2 loops (i.e., greater than 7 glycosylation sites). However, V1V2 length, time since infection and the degree of glycosylation do not reliably predict coreceptor usage. This most likely reflects the complex 3-dimensional nature of the interaction between HIV-1 *env* and the cellular ligands that permit viral attachment and entry.

6. A FINAL LOOK AT STAGE OF ILLNESS

Because of the clear relationship between V1V2 length and stage of illness, we reexamined ***V1V2 length*** vs. ***time since infection*** for different stages separately (Figure S12). Univariate linear regression of length vs. time for each stage separately yielded regression coefficients and coefficients of determination, as follows:

Stage	β	R^2
1	26.9	0.00
2	-2.63	0.11
3	1.17	0.34
4	1.06	0.21

From these data we see from a slightly different perspective how stage strongly influences the dependence of V1V2 length on time since infection. There is clearly no significant relationship for sequences obtained from individuals in stage 1 illness, and a modest *negative* trend over time in individuals with stage 2 illness. This suggests a *regression* in length from randomly transmitted lengths over time after infection, but before chronic illness. In stage 3 samples, we see the strongest trend in both magnitude (β = 1.17) and goodness of fit (R^2 = 0.34) for lengthening over time, and some erosion of this trend for sequences in late stage illness. These data are consistent with selection for short V1V2 loops soon after infection, followed by lengthening under a maturing immune response, followed by regression to shorter (and perhaps competitively more fit) V1V2 loops after relaxation of immune selective pressure in late illness.

7. REANALYSIS OF CHOHAN DATA

Because of the clear relationship between V1V2 sequence length and stage of illness in our data, we reexamined the data presented by Chohan et al., comparing V1V2 sequence length during early and chronic infection with HIV-1 subtype B [9]. Sequences were initially compared as originally presented, and then reexamined after separating sequences from persons with stable chronic infection from individuals meeting AIDS-defining criteria. Sequence lengths were compared using non-parametric methods (Mann-Whitney).

Results: In a strict comparison of the “early” and “chronic + AIDS” groups, we find no difference in V1V2 length (p = 0.11), as originally reported. However, when considered separately, we find significant differences between the “early” and “chronic-stable” groups (p = 0.02), and between the “chronic-stable” and “AIDS” groups (p = 0.01), but not between the “early” and “AIDS” group (p = 0.07)(Figure S13).

Conclusions: Thus these data present a consistent picture of V1V2 lengthening over time, followed by contraction of the V1V2 length during late-stage illness, consistent with our observations in the larger HIV-1 subtype B dataset. There is therefore no inconsistency with early observations on this topic in subtypes A and

C, and based on these data find no need to postulate separate evolutionary mechanisms for HIV-1 subtypes A, B and C.

8. LOOP LENGTH CHANGE DURING TRANSMISSION

Considerable debate exists on the nature of loop length change during transmission of HIV to a new host. While Derdeyn has observed apparent selection for shorter, more neutralization-sensitive V1V4 loops in transmission recipients than in the corresponding donors in subtype C [2], this has not been observed for subtype B by either Frost or Liu [3,4]. We therefore examined loop length variation during transmission for the transmission pairs presented by Frost, Liu, Derdeyn, and a larger set of transmission events involving HIV-1 subtypes A and C presented by Haaland et al [10]. The subtype C portion of the cohort described by Haaland is epidemiologically similar to the cohort presented by Derdeyn, but has the advantage of including a larger number of cases with more accurately known time of transmission. The objectives of these analyses were; 1) to examine loop length changes during transmission in a sufficiently large cohort to draw robust conclusions on changes occurring during transmission; 2) to compare and possibly resolve the differences seen between HIV-1 subtypes A, B and C, and 3) to refine our understanding of which *env* subregions are involved in significant adaptive processes during transmission and early infection.

Data: We obtained all available V1V2 loops corresponding to 44 transmission pairs with evaluable data presented by Derdeyn, Frost, Liu and Haaland (N = 86 individuals, 2300 sequences). Subjects with unknown HIV subtype or unclear transmission epidemiology were excluded (Haaland subjects ZM248M, ZM248F, ZM214M, ZM214F, RW66M, RW66F). For individuals in whom infection was determined by seroconversion between two clinical visits (Derdeyn), time of seroconversion was assumed to occur at the midpoint between last negative and first positive serologies, and the most probable data of infection was assumed to be 42 days prior to estimated date of seroconversion.

Analysis: We calculated the V1V2, V1-V4 and C2-V4 sequence lengths for all sequences in each individual, and plotted **1)** the difference between mean donor and mean recipient sequence length vs time since transmission, and **2)** the difference between mean donor and mean recipient length vs mean donor length. The first plot provides insight into the timing of any selective pressure for longer or shorter loops (i.e. whether selection occurs at transmission or during early infection). If selection for shorter loops occurs at transmission, one would expect a negative trend regardless of the time since infection, whereas if selection occurs as a result of forces acting after transmission in the new host, one might expect a distribution of length changes randomly scattered around zero at early times, and a downward trend at later times.

The second plot provides insight into whether length selection in the newly infected host depends on the length of the transmitted strain (i.e. will variants with short and long sequences both experience selective pressure, or whether there is an optimal sequence length to which newly infecting strains regress in vivo). Here, mean donor length is assumed to reflect the most probable length of the transmitted variant. If selective forces constrain loops to an optimal length, one might expect a downward trend for transmission pairs in which the donor has relatively long loops, and little change in the case of donors with short loops. If no such forces are present, one might expect values randomly distributed around 0.

Results: Transmission pairs were sampled at a mean of 0.16 years post transmission (range 0.38 to 0.58 years). However, all pairs except for one were sampled at times < 0.3 years. 27 transmission pairs showed a decline in mean V1V2 length, while either no change or a mean length increase was seen in the remaining 17 pairs. Similarly, decreases in V1V4 and C2V4 were seen in 18 pairs. The mean change in V1V2 length was -1.7 amino acids (range + 8.6 to -15.6). Much of the variation in total V1V4 length occurring across transmission can be attributed to the V1V2 loop, while length change from C2-V4 was modest (Figure 14, panels A-C). While a trend towards decreasing V1V2 loop length was seen for the pairs presented by Derdeyn (5 of 8), a consistent trend was difficult to see among all subtype C pairs combined, in subtypes A or B, or in the dataset as a whole (Figure 14, panel A). Similar results were obtained when using median values (data not shown). Interestingly, in an examination of loop change as a function of donor length, we see a modestly significant downward trend for V1V2 sequences, but no significant trends for the other regions (Figure 14, panel D). Regression values for these regions were:

Region	β	R^2
V1V2	-0.23	0.12
C2-V4	0.07	0.01
V1V4	-0.10	0.02

Conclusions: In these data, it is difficult to see a clear trend towards increasing or decreasing loop length after transmission. One limitation of this analysis is that the available data do not provide an opportunity to examine loop length change at times > 0.3 years post infection, when declines in loop length might be predicted to occur as a result of selection at times after the immediate post-infection period, as suggested in our cross-sectional analyses. It is worth noting that time since infection was known with somewhat less precision in the sequences described by Derdeyn than in those by Haaland, Frost and Liu, and it may be that these sequences were sampled at later times than estimated. If so, a declining trend might be expected in these sequences, in contrast to those presented by Haaland. Overall, the data presented here suggest no length selection in any region at the time of selection, and possible selection for shorter V1V2 loops at times after 0.3 years in cases where the transmitted variant exceeds a length of 60 amino acids.

Citations

1. Jensen MA, Li FS, van 't Wout AB, Nickle DC, Shriner D, et al. (2003) Improved coreceptor usage prediction and genotypic monitoring of R5-to-X4 transition by motif analysis of human immunodeficiency virus type 1 env V3 loop sequences. J Virol 77: 13376-13388.
2. Derdeyn CA, Decker JM, Bibollet-Ruche F, Mokili JL, Muldoon M, et al. (2004) Envelope-constrained neutralization-sensitive HIV-1 after heterosexual transmission. Science 303: 2019-2022.
3. Frost SD, Liu Y, Pond SL, Chappey C, Wrin T, et al. (2005) Characterization of human immunodeficiency virus type 1 (HIV-1) envelope variation and neutralizing antibody responses during transmission of HIV-1 subtype B. J Virol 79: 6523-6527.
4. Liu Y, Curlin ME, Diem K, Zhao H, Ghosh AK, et al. (2008) Env length and N-linked glycosylation following transmission of human immunodeficiency virus Type 1 subtype B viruses. Virology 374: 229-233.
5. Pillai S, Good B, Richman D, Corbeil J (2003) A new perspective on V3 phenotype prediction. AIDS Res Hum Retroviruses 19: 145-149.
6. Lengauer T, Sander O, Sierra S, Thielen A, Kaiser R (2007) Bioinformatics prediction of HIV coreceptor usage. Nat Biotechnol 25: 1407-1410.
7. Boisvert S, Marchand M, Laviolette F, Corbeil J (2008) HIV-1 coreceptor usage prediction without multiple alignments: an application of string kernels. Retrovirology 5: 110.
8. Shankarappa R, Margolick JB, Gange SJ, Rodrigo AG, Upchurch D, et al. (1999) Consistent viral evolutionary changes associated with the progression of human immunodeficiency virus type 1 infection. J Virol 73: 10489-10502.
9. Chohan B, Lang D, Sagar M, Korber B, Lavreys L, et al. (2005) Selection for human immunodeficiency virus type 1 envelope glycosylation variants with shorter V1-V2 loop sequences occurs during transmission of certain genetic subtypes and may impact viral RNA levels. J Virol 79: 6528-6531.
10. Haaland RE, Hawkins PA, Salazar-Gonzalez J, Johnson A, Tichacek A, et al. (2009) Inflammatory genital infections mitigate a severe genetic bottleneck in heterosexual transmission of subtype A and C HIV-1. PLoS Pathog 5: e1000274.
11. Zolla-Pazner S (2004) Identifying epitopes of HIV-1 that induce protective antibodies. Nat Rev Immunol 4: 199-210.
