# Supplementary figures and images for: HIV-1 Envelope Subregion Length Variation during Disease Progression
Source: PLoS Pathog. 2010 Dec 16;6(12):e1001228. doi: 10.1371/journal.ppat.1001228 (PMC3002983; doi:10.1371/journal.ppat.1001228)

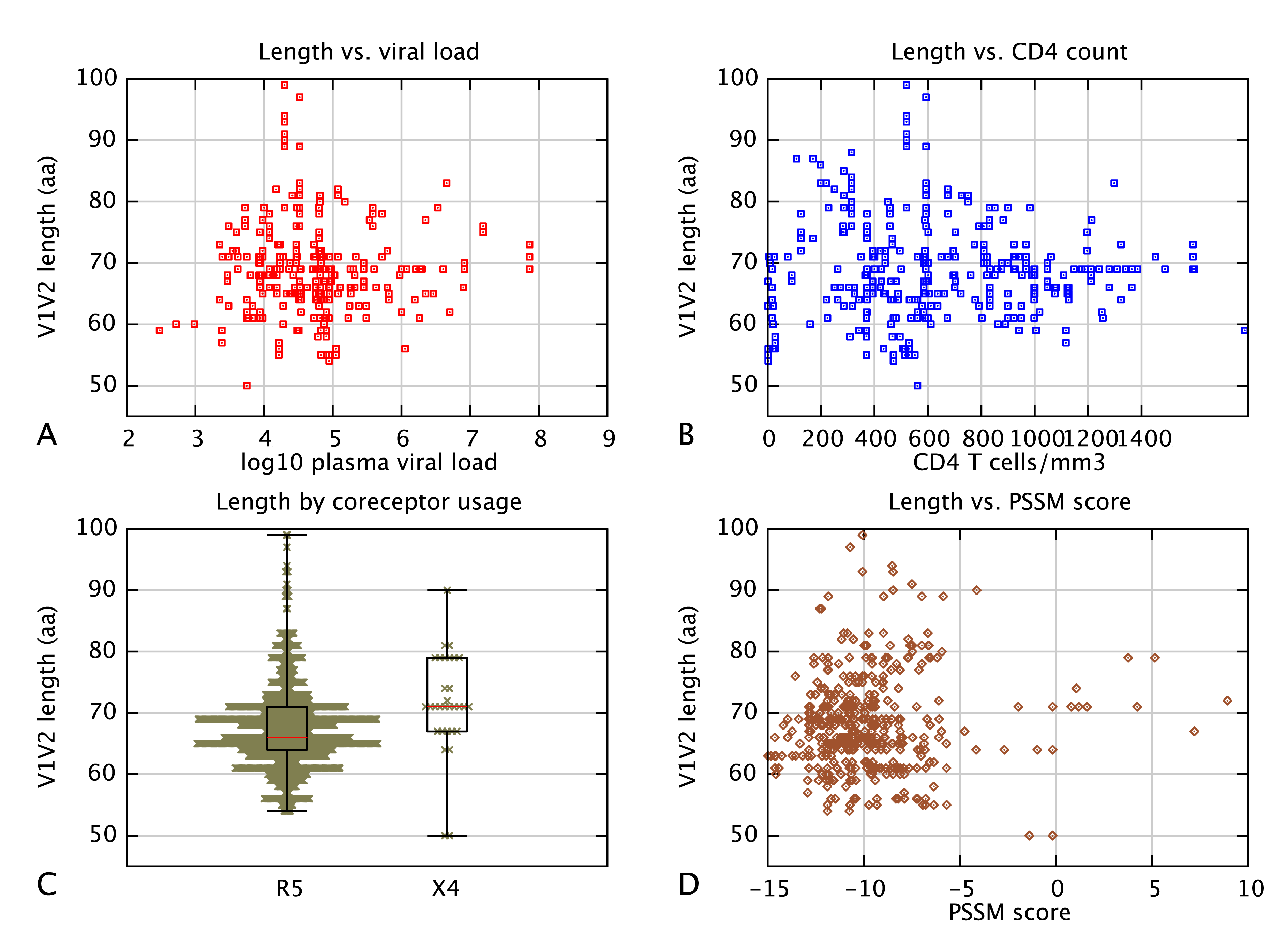

Supplement: Figure S1 — V1V2 length vs. virologic and clinical parameters I. Panel A: V1V2 length vs. log10 plasma viral load (no significant relationship). Panel B: V1V2 length vs. peripheral CD4 T-cell count (no significant relationship). Panel C: V1V2 length by coreceptor usage. Box-plots depict minimum, 1st quartile, median (red line), 3rd quartile and maximum values in each group, with superimposed individual length measurements. In this series, V1V2 sequences associated with V3 loops predicted to be X4-tropic by PSSM are slightly longer compared with sequences associated with R5-tropic V3 loops (median 71 vs. 66 amino acids, p = 3.49×10^-5^, MW test). However, a plot of V1V2 length vs. PSSM score (Panel D) does not reveal a clear linear correlation between V1V2 length and PSSM score. (1.15 MB TIF) [file ppat.1001228.s002.tif]

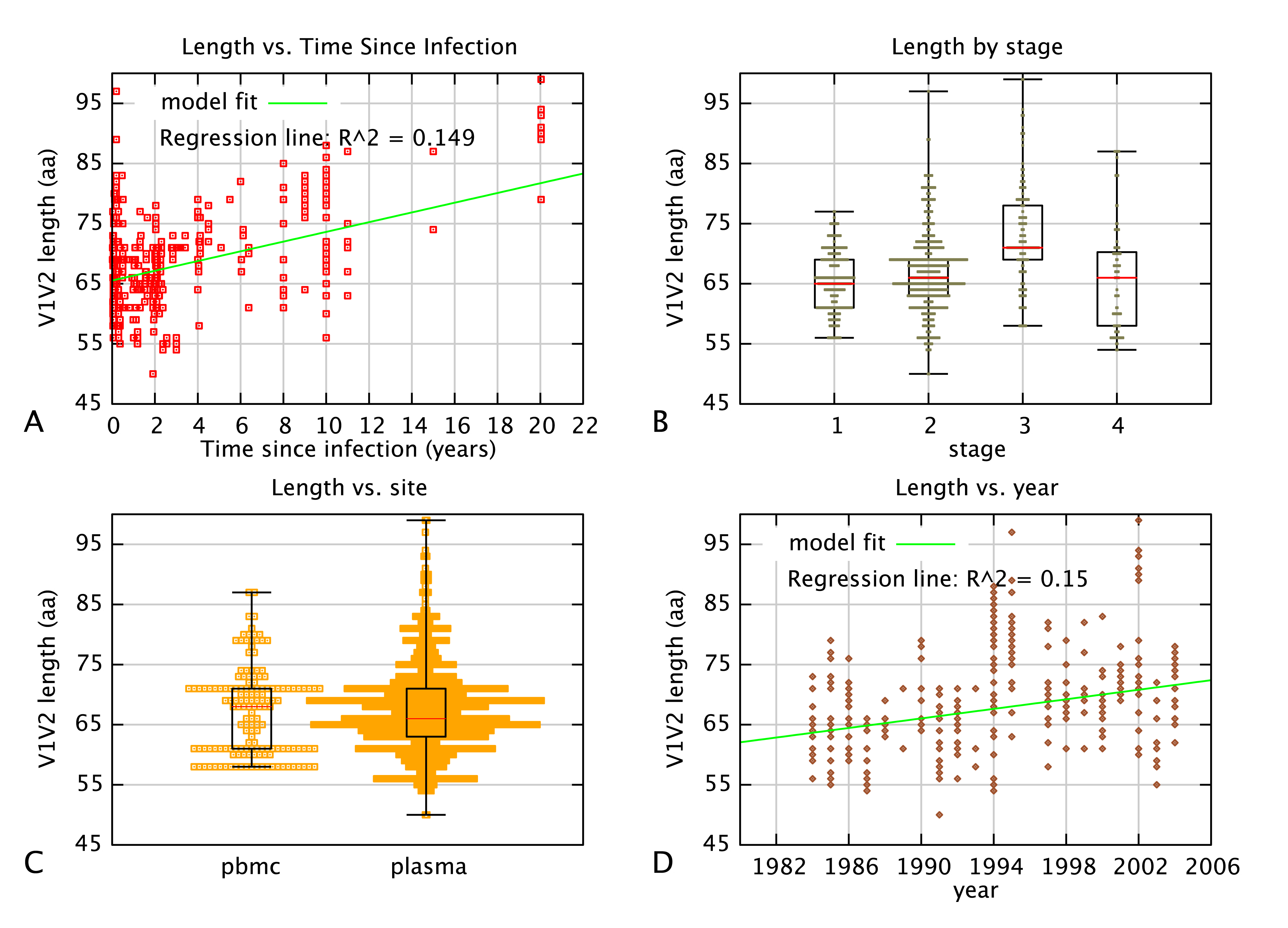

Supplement: Figure S2 — V1V2 length vs. virologic and clinical parameters II. Panel A: V1V2 length vs. time since infection. As described earlier, a significant positive correlation V1V2 length and time since infection is evident (R = 0.149) Panel B: V1V2 length by stage of infection. Box-plots depict minimum, 1st quartile, median (red line), 3rd quartile and maximum values in each stage group, with superimposed individual length measurements. Highly significant differences in V1V2 length are seen between stage 3 and stages 1,2 and 4 (p<2.2×10^−16^, M-W rank sum test), reflecting V1V2 lengthening in chronic illness, followed by contraction in late disease. Panel C: V1V2 length by site (PBMC vs. plasma). In this univariate comparison, there is no significant length difference between V1V2 loops obtained from PBMC (median 68 amino acids) and plasma (median 66 amino acids, p = 0.93). Panel D: V1V2 length vs. year of sampling. As described, there is a significant positive correlation between V1V2 length and year of sampling. (1.14 MB TIF) [file ppat.1001228.s003.tif]

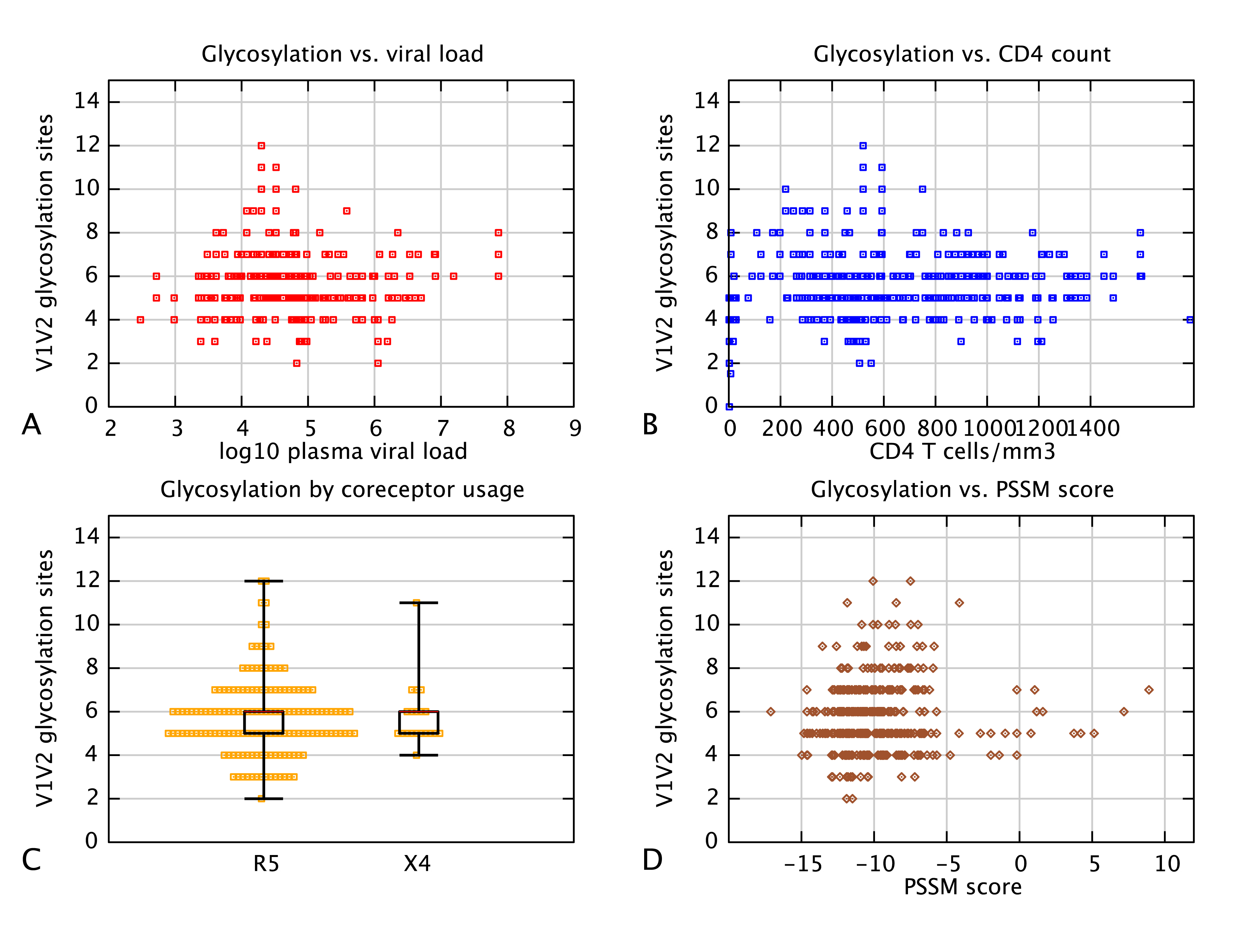

Supplement: Figure S3 — V1V2 glycosylation vs. virological and clinical parameters I. Panel A: Number of V1V2 glycosylation sites vs. log10 plasma viral load (no significant relationship). Panel B: Number of V1V2 glycosylation sites vs. peripheral CD4 T-cell count (no clear correlation observed). Panel C: V1V2 glycosylation sites by inferred coreceptor usage (R5 or X4). Box-plots report minimum, 1st quartile, median (red line), 3rd quartile and maximum values in each stage group, with superimposed individual measurements. No clear differences in glycosylation are noted between V1V2 loops associated with R5-tropic and X4-tropic V3 loops (median 6 and 6 PNLGS, respectively). Panel D: Number of V1V2 glycosylation sites vs. PSSM score (no significant relationship). (1.07 MB TIF) [file ppat.1001228.s004.tif]

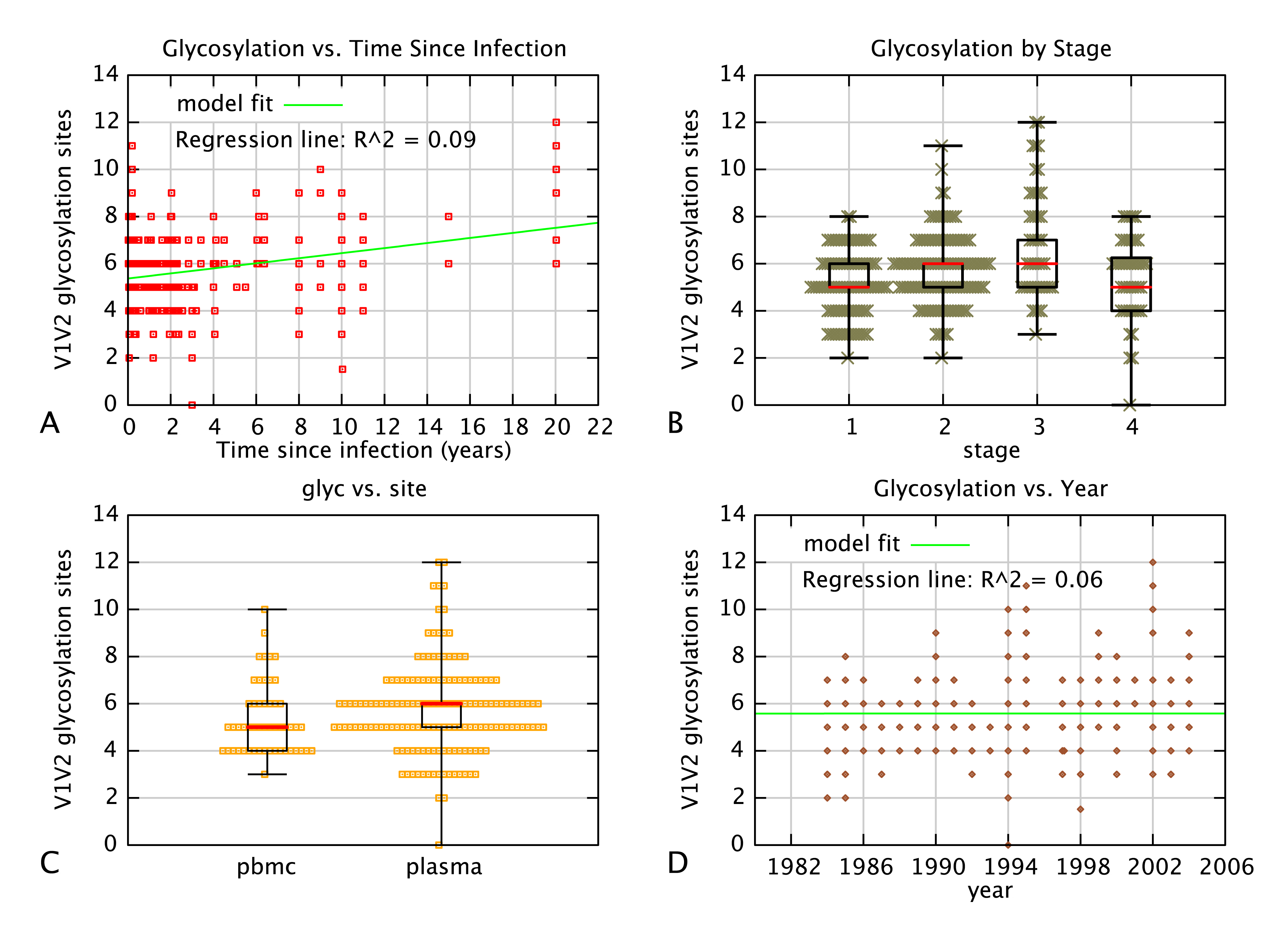

Supplement: Figure S4 — V1V2 glycosylation vs. virological and clinical parameters II. Panel A: Number of V1V2 glycosylation sites vs. time since infection. As with V1V2 length, in a univariate analysis there is a modest but significant linear correlation between time since infection and the extent of V1V2 glycosylation (β = 0.12 amino acids/year, R2 = 0.09). Panel B: Number of V1V2 glycosylation sites by clinical stage. Similar to what was observed for V1V2 length, glycosylation in chronic illness (stage 3) was significantly greater than in early and late disease (p<1×10^−8^), reflecting increasing glycosylation during chronic infection, followed by a decline in the extent of glycosylation during AIDS. Panel C: V1V2 glycosylation sites by site (PBMC or plasma). Box-plots report minimum, 1st quartile, median (red line), 3rd quartile and maximum values in each stage group, with superimposed individual measurements. No clear differences in glycosylation are noted between V1V2 loops obtained from PBMC vs. plasma (median PNLGs 5 and 6, respectively, p = 0.59). Panel D: Number of V1V2 glycosylation sites vs. year of sampling. There is a negligible positive correlation between V1V2 PNLG and year of sampling (β = 0.05, R2 = 0.06) (1.13 MB TIF) [file ppat.1001228.s005.tif]

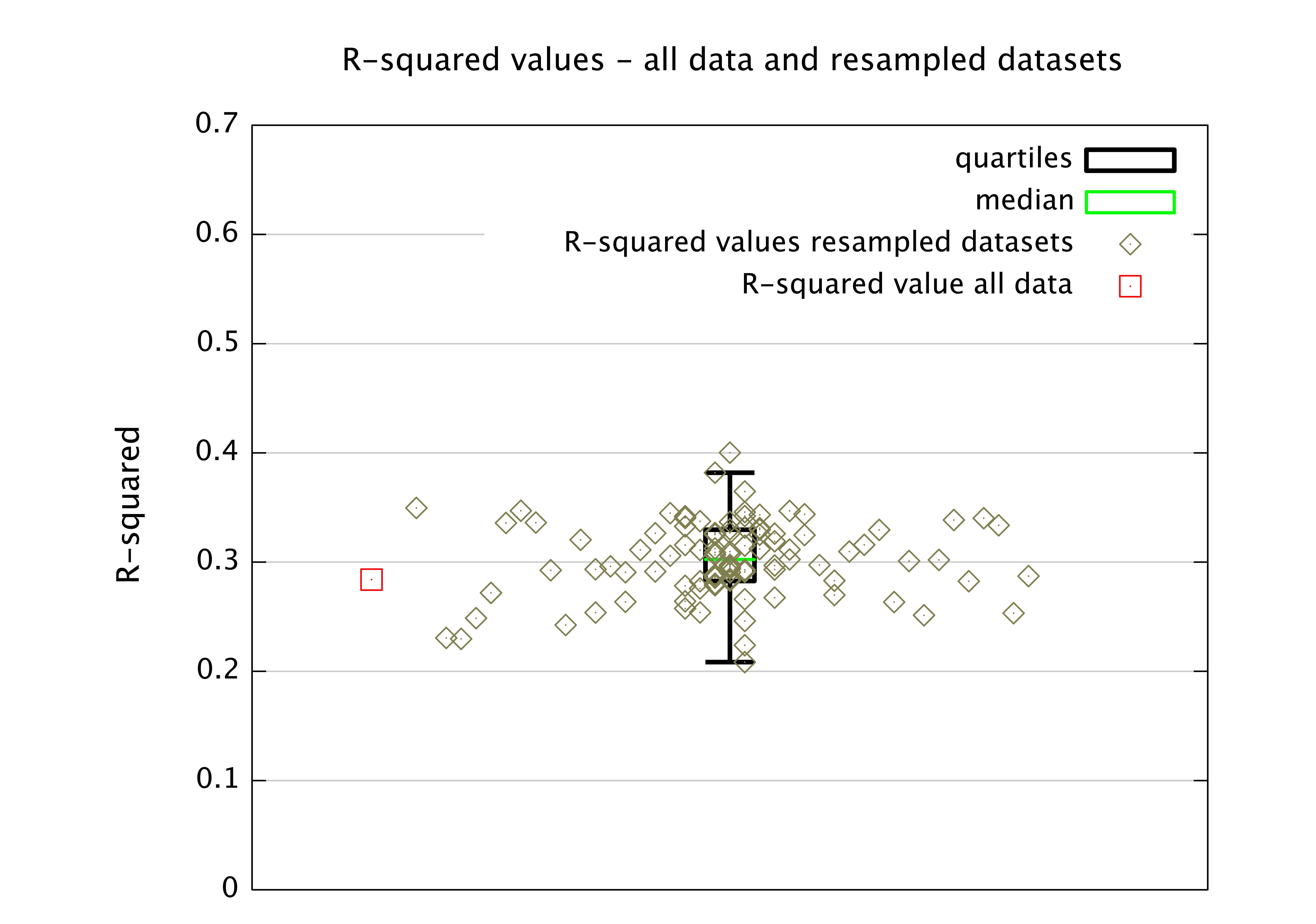

Supplement: Figure S5 — Resampling analysis: R2 values for multiple linear regression of V1V2 length on the independent variables time since infection, year of sampling, and sample type for the entire dataset (red squares □) and for 100 parallel randomly resampled datasets derived from the original dataset (green diamonds ⋄). Correlation coefficients obtained in the resampled datasets were consistent with the correlation coefficient obtained using all data. (1.33 MB TIF) [file ppat.1001228.s006.tif]

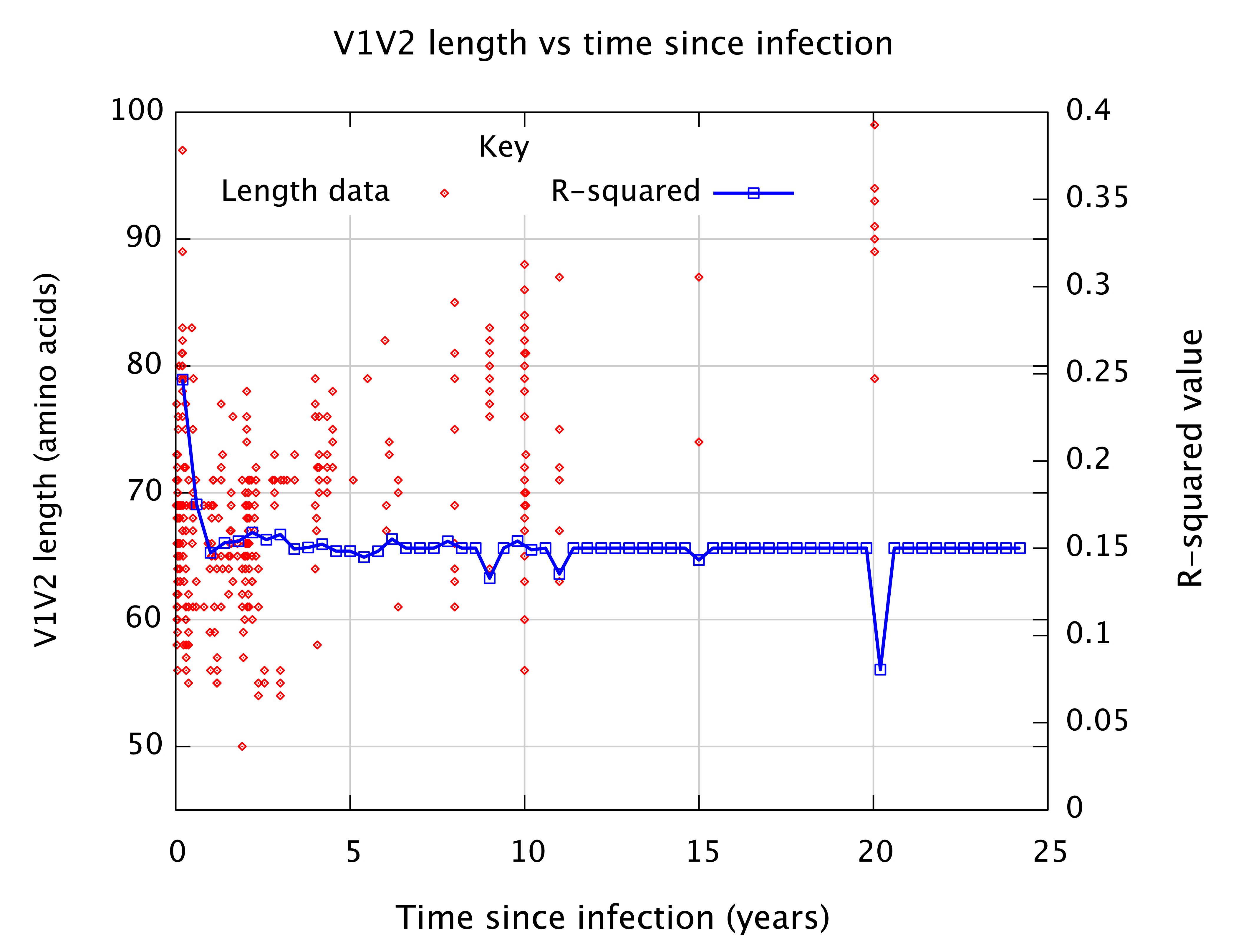

Supplement: Figure S6 — V1V2 sequence length vs. time since infection - sliding window analysis: Length measurements (red + sign) and R2 values (blue triangles Δ) for univariate linear regression analyses of datasets excluding 0.4-year periods since the time of infection. 0.4-year data exclusion periods are centered around the x value of each Δ datapoint. The correlation strength of the linear model is greatest for datasets excluding the earliest two 0.4-year periods (first two datapoints), indicating that linear regression of V1V2 length on time since infection most accurately explains data obtained at times after approximately 0.8 years. (1.07 MB TIF) [file ppat.1001228.s007.tif]

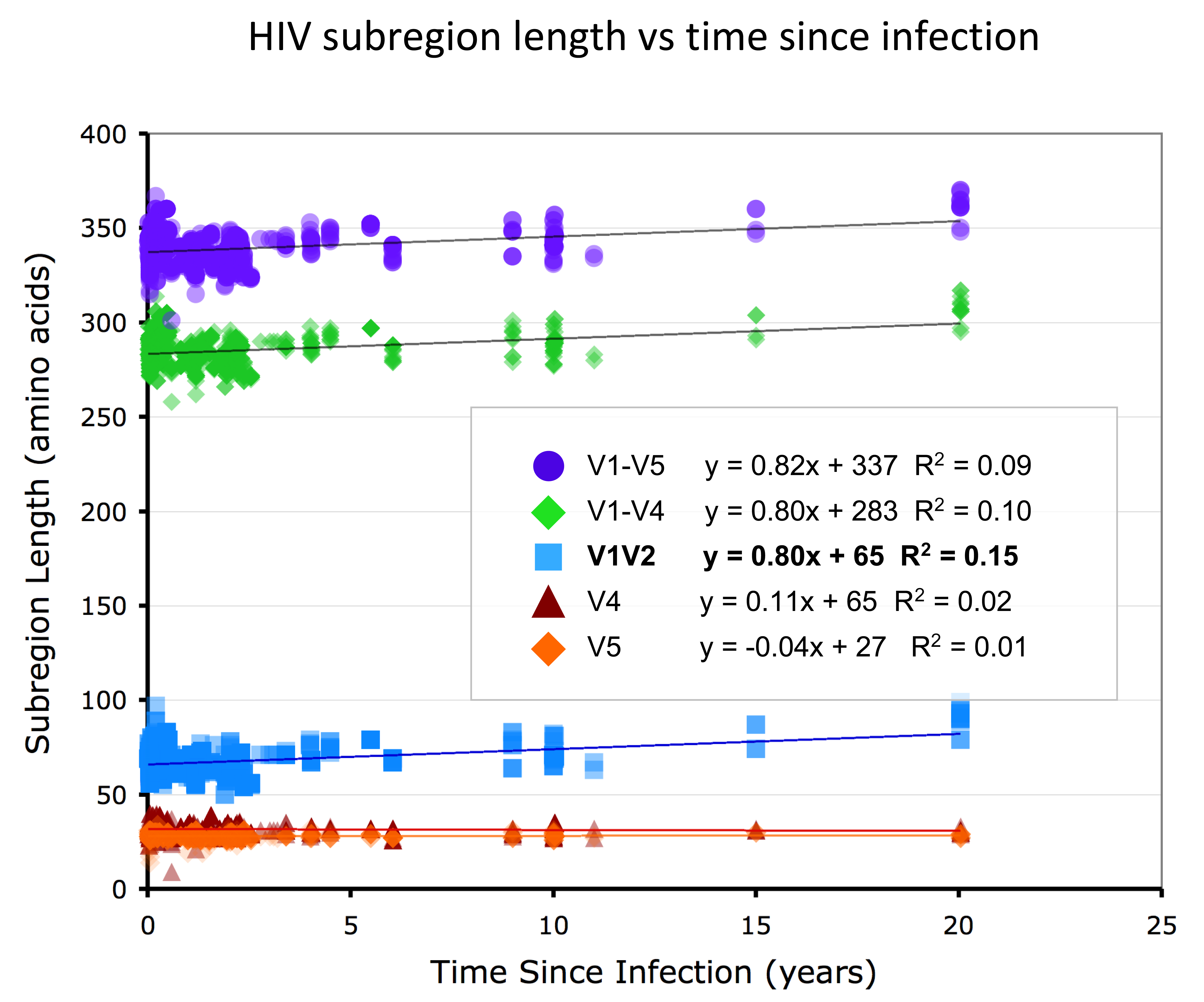

Supplement: Figure S7 — Subregion length (V1–V5) vs. time since infection for the V1V5 region (purple circles), the V1V4 region (green diamonds) and V1V2 (blue squares), V4 (red triangles) and V5 (orange diamonds) considered separately. A significant trend towards increasing length seen in V1V2, V1V4 and V1V5 can be ascribed primarily to changes in V1V2. (2.13 MB TIF) [file ppat.1001228.s008.tif]

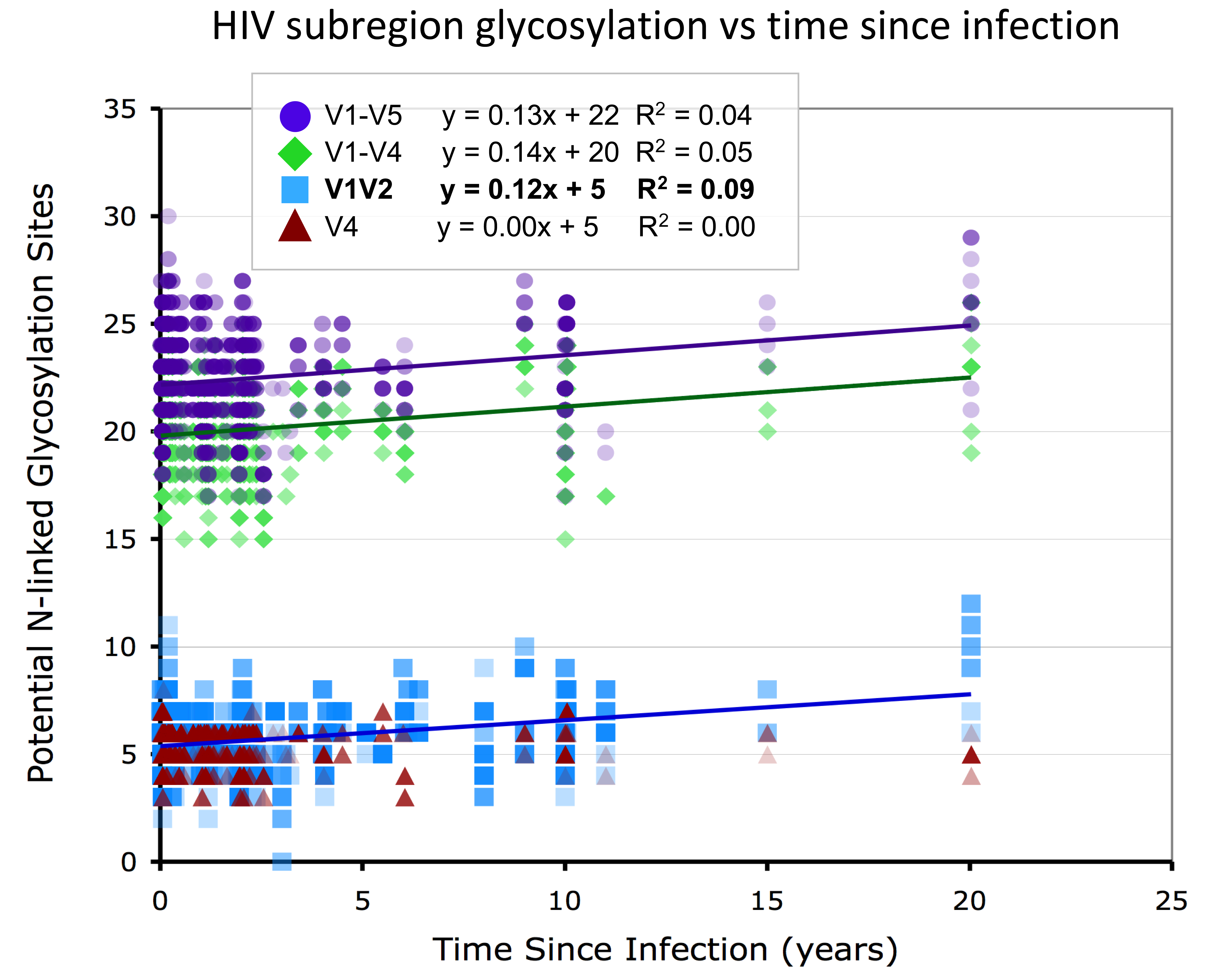

Supplement: Figure S8 — Subregion glycosylation (V1–V5) vs. time since infection for the V1V5 region (purple circles), the V1V4 region (green diamonds) and for V1V2 (blue squares) and V4 (red triangles) considered separately. A modest trend towards increasing glycosylaton seen in V1V2, V1V4 and V1V5 can be ascribed primarily to changes in V1V2 (2.38 MB TIF) [file ppat.1001228.s009.tif]

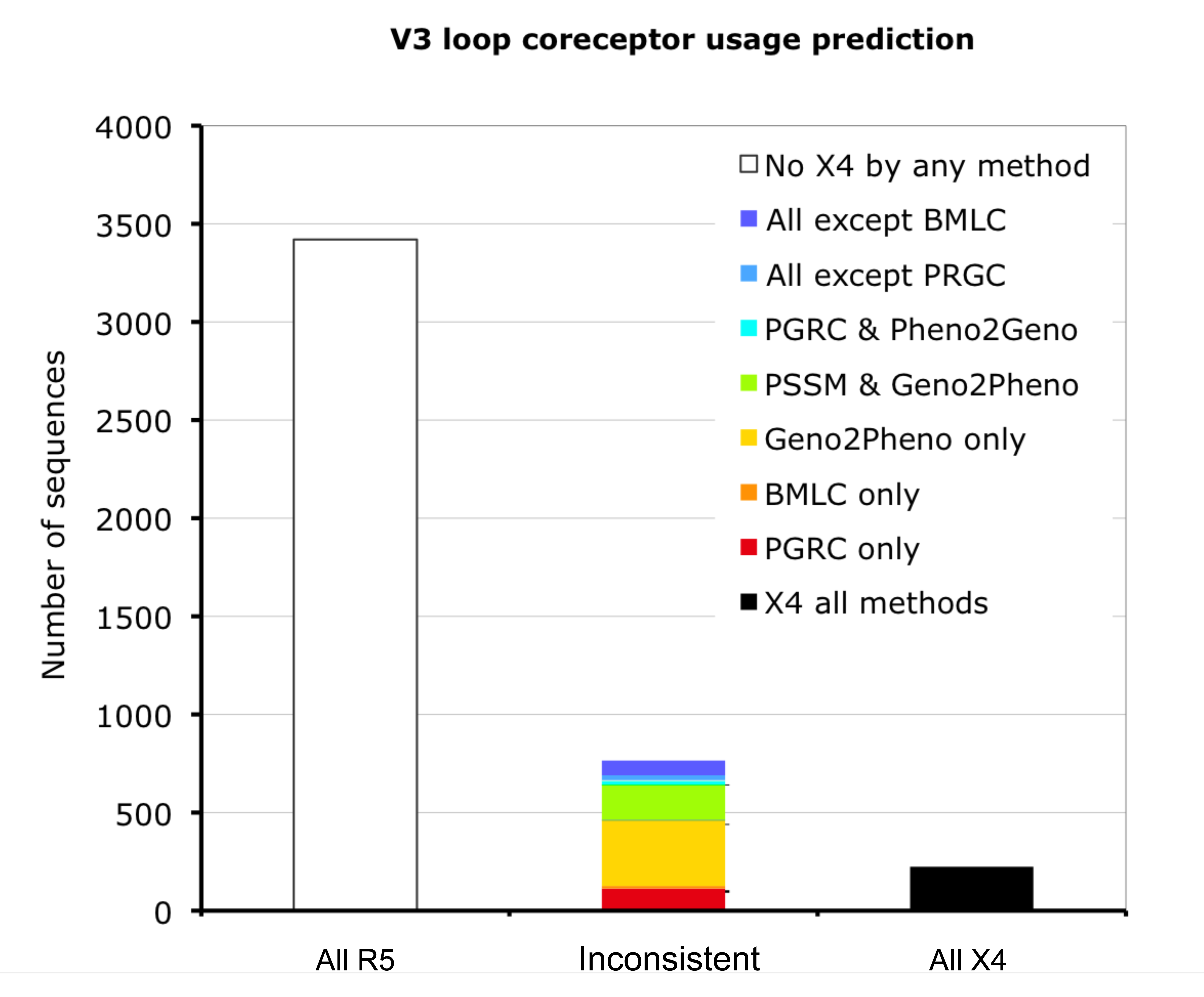

Supplement: Figure S9 — Agreement between 4 bioinformatic coreceptors used to assign probable coreceptor usage. There was complete agreement between all methods for ∼80% of sequences examined, while in the remaining 20%, there was some disagreement in assignment between one or more scoring methods. Most sequences were predicted to be CCR5-tropic by all methods (white bar), while a modest number of sequences was predicted to be CXCR4-tropic by all methods. The remaining sequences were scored differently by various methods, as represented (colored bars). (2.84 MB TIF) [file ppat.1001228.s010.tif]

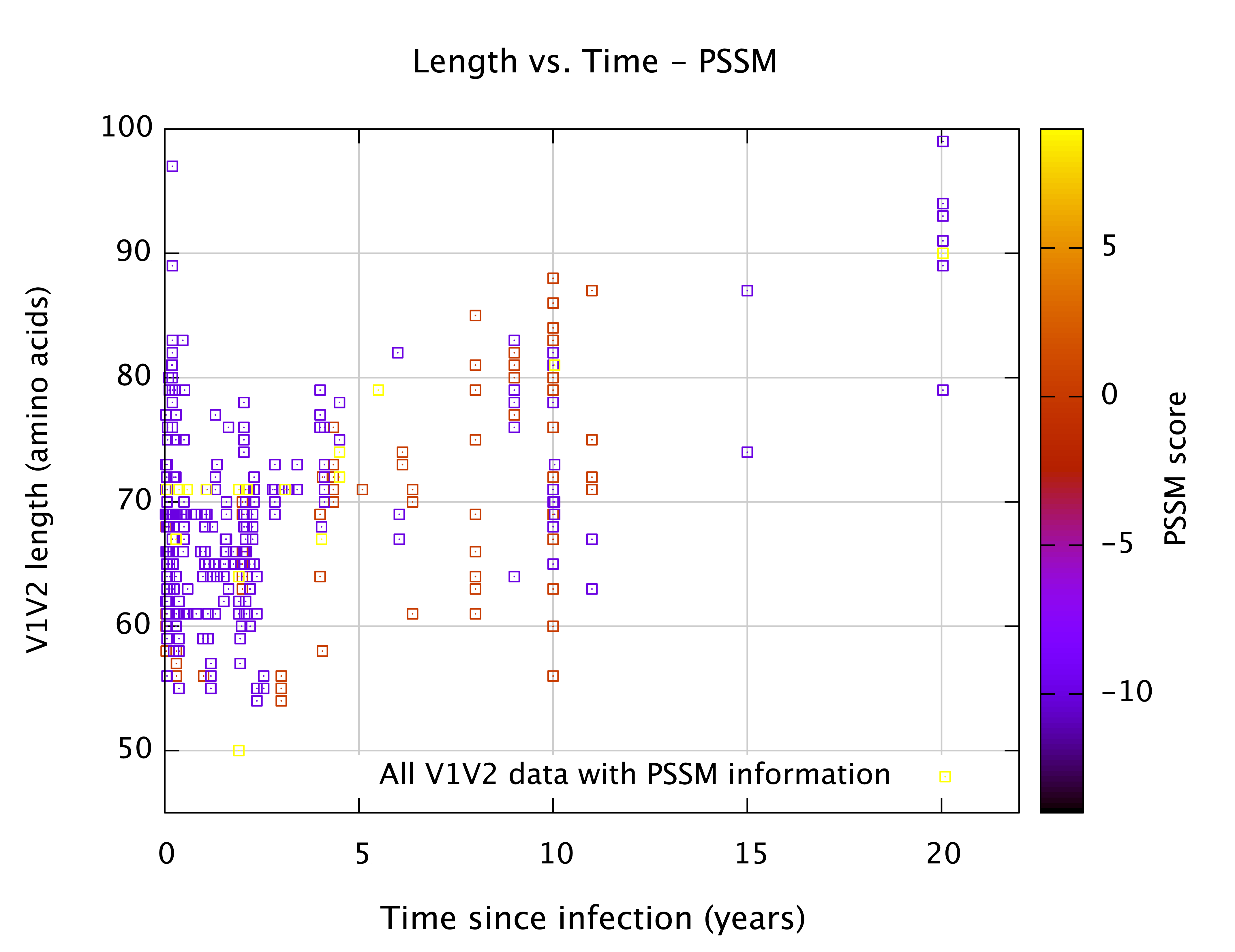

Supplement: Figure S10 — V1V2 sequence length vs. time since infection and PSSM score. Rising PSSM scores (color scale), depicted as warmer colors, indicate a greater likelihood of CXCR4 coreceptor usage; in this dataset, predicted X4 coreceptor usage occurs at a PSSM score of approximately -2. In these data, there is a pronounced preponderance of CCR5-using viruses, with a trend towards increasing prevalence of X4-tropic viruses during chronic infection. However, X4 and R5 viruses are distributed throughout all infection times, and cannot be easily distinguished on the basis of V1V2 length. (1.15 MB TIF) [file ppat.1001228.s011.tif]

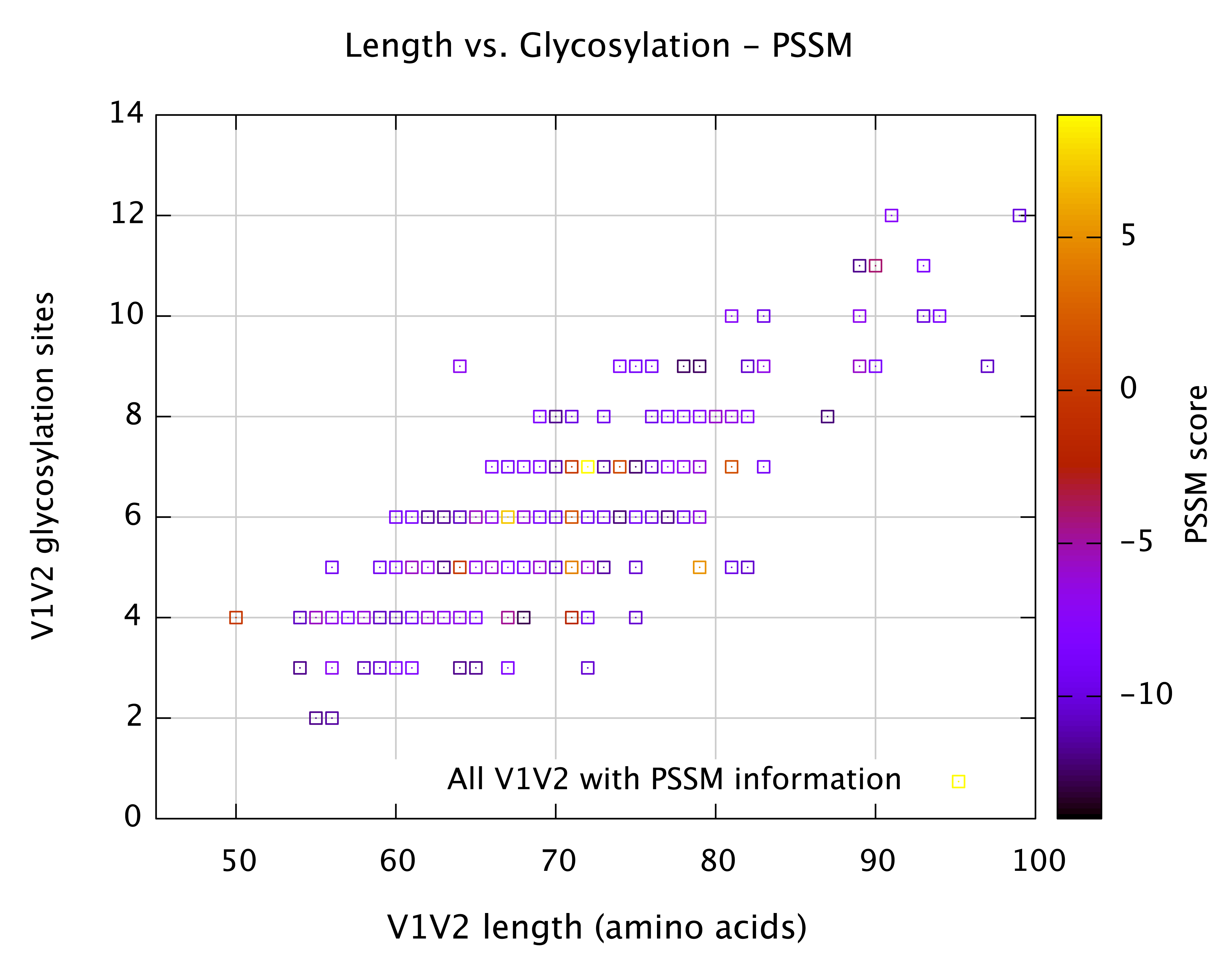

Supplement: Figure S11 — V1V2 potential N-linked glycosylation sites vs. V1V2 length and PSSM score (color scale). There is a very marked dependence of glycosylation on length (β = 0.13 PNGL/amino acid, R2 = 0.52). X4-usage appears to be more commonly associated with V1V2 sequences bearing 4-7 PNLG sites, than with sequences with more than 7 sites (and see figure S1 panel D). (1.10 MB TIF) [file ppat.1001228.s012.tif]

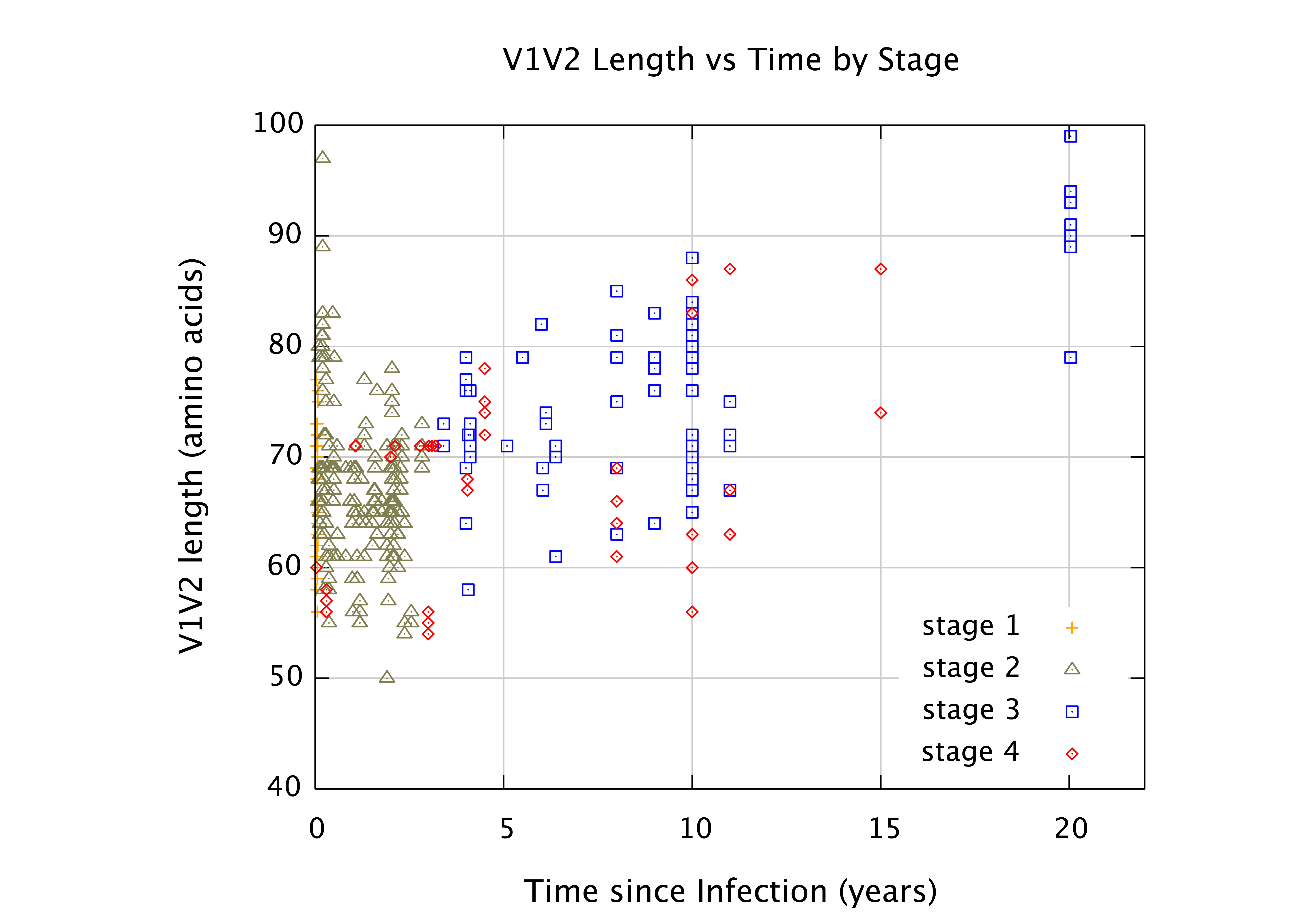

Supplement: Figure S12 — V1V2 and Stage of Illness. V1V2 length vs. Time since Infection for stage 1 (orange “+”), stage 2 (gray triangles), stage 3 (blue squares), and stage 4 (red diamonds). There is a slight decline in V1V2 length from stage 1 to stage 2, reflecting regression from transmitted viruses of essentially random lengths to shorter loop lengths during early infection prior to the onset of a meaningful immune response. This is followed by a strong trend towards lengthening during chronic infection (stage 3) and a weakening of this trend in late-stage illness (stage 4). (1.52 MB TIF) [file ppat.1001228.s013.tif]

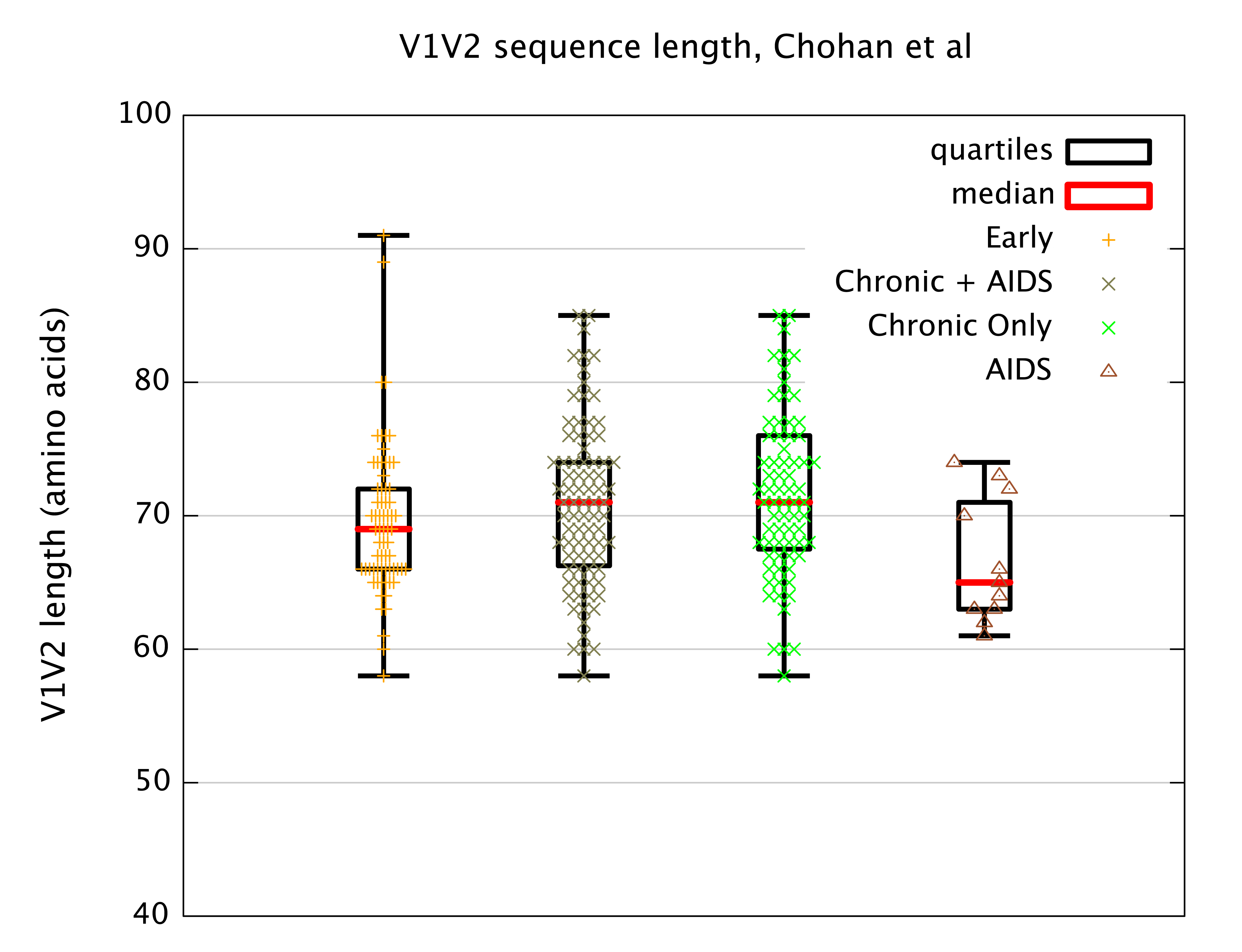

Supplement: Figure S13 — Chohan Data revisited: V1V2 sequence length for subjects in early infection (first bar), chronic infection and AIDS considered together (second bar), chronic stable infection only (third bar), and individuals with AIDS-defining clinical conditions (fourth bar). Length differences between “early”, “chronic” and “AIDS” are statistically significant (p≤0.02). Thus, separation of sequences obtained during AIDS from sequences obtained during chronic stable infection reveals a trend of rising V1V2 length through chronic infection, followed by falling length in AIDS that is not otherwise apparent. (1.04 MB TIF) [file ppat.1001228.s014.tif]

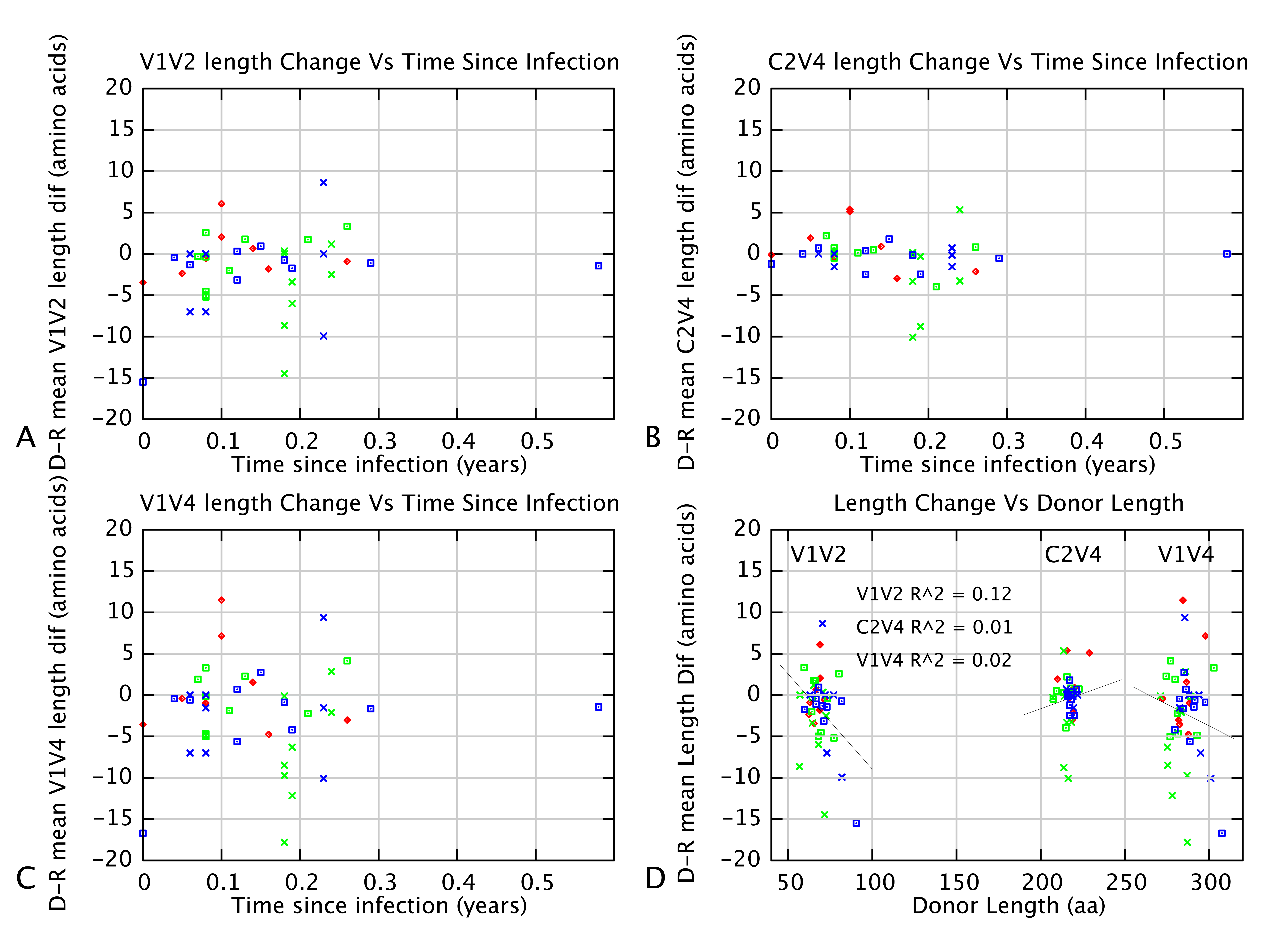

Supplement: Figure S14 — V1V2 length during transmission: Change in mean loop length between donor and recipient in 44 transmission pairs involving HIV-1 subtypes A, C and B, presented by Haaland, Derdeyn, Frost and Liu. Panels A–C: Difference in mean loop length between donors and recipients vs. time since infection for V1V2 (panel A), C2–V4 (panel B), and V1–V4 (panel C). Panel D: Difference in mean loop length between donors and recipients vs. the mean loop length (for the corresponding region) in the donor. Subtype A sequences (Haaland, represented by red +), Subtype B sequences (Frost, blue X) and Liu (blue squares) and subtype C sequences (Haaland, green squares, and Derdeyn, green X). (2.25 MB TIF) [file ppat.1001228.s015.tif]
