# Supplementary material for: HIV-1 Envelope Subregion Length Variation during Disease Progression
Source: PLoS Pathog. 2010 Dec 16;6(12):e1001228. doi: 10.1371/journal.ppat.1001228 (PMC3002983; doi:10.1371/journal.ppat.1001228)
Supplement: Table S1 — Published sequence data. Accession Numbers for previously published sequences included in cross-sectional, longitudinal and transmission analyses. (0.05 MB PDF) [file ppat.1001228.s016.pdf]

Author	Ref	Accession Numbers
Aasa-Chapman	[1]	AY295200-12, 22-26, 33-38
Bagnarelli	[2]	AF105432-5961
Bello	[3]	AY497912-8031
Dacheux	[4]	AY535425-518
Daniels	[5]	AJ535590-619
Delwart	[6]	AF403510-38
Derdeyn	[7]	AY423908-4198
Fang	[8]	U69584-93
Frost	[9]	Obtained directly from Authors
Haaland	[11]	FJ185853-7678
Hayman	[12]	AY428618-20
Kuhmann	[13]	AY357345, AY357515-580
McDonald	[14]	U69282-391, U69396, U69401-481
Poss	[15]	AF047981- 8284, -8523, -8536-44, -69, -73, -75-8, -591, 598-603, 611, 656, 668-9
Quiñones-Mateu	[16]	U16764, U16772-778
Shioda	[17]	AB002829-2974, -2988-3019
Spudich	[18]	AY842796-2807, AY842815-23
Wei	[19]	AY223719- AY223790
Yi	[20]	DQ853426-DQ853435, DQ853455-DQ853464, EU588732-EU588968
Zheng	[21]	AJ418478- AJ418530
Zhu	[22]	L21224, L21234-302

Table S1. **Published sequence data.** Accession Numbers for previously published sequences included in cross-sectional, longitudinal and transmission analyses.

References

1. Aasa-Chapman MM, Hayman A, Newton P, Cornforth D, Williams I, et al. (2004) Development of the antibody response in acute HIV-1 infection. AIDS 18: 371-381.
2. Bagnarelli P, Mazzola F, Menzo S, Montroni M, Butini L, et al. (1999) Host-specific modulation of the selective constraints driving human immunodeficiency virus type 1 env gene evolution. J Virol 73: 3764-3777.
3. Bello G, Casado C, Garcia S, Rodriguez C, del Romero J, et al. (2004) Co-existence of recent and ancestral nucleotide sequences in viral quasispecies of human immunodeficiency virus type 1 patients. J Gen Virol 85: 399-407.
4. Dacheux L, Moreau A, Ataman-Onal Y, Biron F, Verrier B, et al. (2004) Evolutionary dynamics of the glycan shield of the human immunodeficiency virus envelope during natural infection and implications for exposure of the 2G12 epitope. J Virol 78: 12625-12637.
5. Daniels RS, Wilson P, Patel D, Longhurst H, Patterson S (2004) Analysis of full-length HIV type 1 env genes indicates differences between the virus infecting T cells and dendritic cells in peripheral blood of infected patients. AIDS Res Hum Retroviruses 20: 409-413.
6. Delwart E, Magierowska M, Royz M, Foley B, Peddada L, et al. (2002) Homogeneous quasispecies in 16 out of 17 individuals during very early HIV-1 primary infection. AIDS 16: 189-195.
7. Derdeyn CA, Decker JM, Bibollet-Ruche F, Mokili JL, Muldoon M, et al. (2004) Envelope-constrained neutralization-sensitive HIV-1 after heterosexual transmission. Science 303: 2019-2022.
8. Fang G, Burger H, Chappey C, Rowland-Jones S, Visosky A, et al. (2001) Analysis of transition from long-term nonprogressive to progressive infection identifies sequences that may attenuate HIV type 1. AIDS Res Hum Retroviruses 17: 1395-1404.
9. Frost SD, Liu Y, Pond SL, Chappey C, Wrin T, et al. (2005) Characterization of human immunodeficiency virus type 1 (HIV-1) envelope variation and neutralizing antibody responses during transmission of HIV-1 subtype B. J Virol 79: 6523-6527.
10. Gao F, Morrison SG, Robertson DL, Thornton CL, Craig S, et al. (1996) Molecular cloning and analysis of functional envelope genes from human immunodeficiency virus type 1 sequence subtypes A through G. The WHO and NIAID Networks for HIV Isolation and Characterization. J Virol 70: 1651-1667.
11. Haaland RE, Hawkins PA, Salazar-Gonzalez J, Johnson A, Tichacek A, et al. (2009) Inflammatory genital infections mitigate a severe genetic bottleneck in heterosexual transmission of subtype A and C HIV-1. PLoS Pathog 5: e1000274.

12. Hayman A, Moss T, Simmons G, Arnold C, Holmes EC, et al. (2001) Phylogenetic analysis of multiple heterosexual transmission events involving subtype b of HIV type 1. AIDS Res Hum Retroviruses 17: 689-695.
13. Kuhmann SE, Pugach P, Kunstman KJ, Taylor J, Stanfield RL, et al. (2004) Genetic and phenotypic analyses of human immunodeficiency virus type 1 escape from a small-molecule CCR5 inhibitor. J Virol 78: 2790-2807.
14. McDonald RA, Mayers DL, Chung RC, Wagner KF, Ratto-Kim S, et al. (1997) Evolution of human immunodeficiency virus type 1 env sequence variation in patients with diverse rates of disease progression and T-cell function. J Virol 71: 1871-1879.
15. Poss M, Rodrigo AG, Gosink JJ, Learn GH, de Vange Panteleeff D, et al. (1998) Evolution of envelope sequences from the genital tract and peripheral blood of women infected with clade A human immunodeficiency virus type 1. J Virol 72: 8240-8251.
16. Quinones-Mateu ME, Holguin A, Dopazo J, Najera I, Domingo E (1996) Point mutant frequencies in the pol gene of human immunodeficiency virus type 1 are two- to threefold lower than those of env. AIDS Res Hum Retroviruses 12: 1117-1128.
17. Shioda T, Oka S, Xin X, Liu H, Harukuni R, et al. (1997) In vivo sequence variability of human immunodeficiency virus type 1 envelope gp120: association of V2 extension with slow disease progression. J Virol 71: 4871-4881.
18. Spudich SS, Huang W, Nilsson AC, Petropoulos CJ, Liegler TJ, et al. (2005) HIV-1 chemokine coreceptor utilization in paired cerebrospinal fluid and plasma samples: a survey of subjects with viremia. J Infect Dis 191: 890-898.
19. Wei X, Decker JM, Wang S, Hui H, Kappes JC, et al. (2003) Antibody neutralization and escape by HIV-1. Nature 422: 307-312.
20. Liu Y, Curlin ME, Diem K, Zhao H, Ghosh AK, et al. (2008) Env length and N-linked glycosylation following transmission of human immunodeficiency virus Type 1 subtype B viruses. Virology 374: 229-233.
21. Zheng NN, Vella C, Easterbrook PJ, Daniels RS (2002) Selection following isolation of human immunodeficiency virus type 1 in peripheral blood mononuclear cells and herpesvirus saimiri-transformed T cells is comparable. J Gen Virol 83: 1343-1352.
22. Zhu T, Mo H, Wang N, Nam DS, Cao Y, et al. (1993) Genotypic and phenotypic characterization of HIV-1 patients with primary infection. Science 261: 1179-1181.
