# Supplementary material for: HIV-1 Envelope Subregion Length Variation during Disease Progression
Source: PLoS Pathog. 2010 Dec 16;6(12):e1001228. doi: 10.1371/journal.ppat.1001228 (PMC3002983; doi:10.1371/journal.ppat.1001228)
Supplement: Table S2 — Multivariable regression analysis of V1V2 length vs. clinical variables, upper and lower 5% excluded. Beta coefficients for V1V2 Length vs. Time since Infection (Model 1), Stage of Infection (Model 2), CD4 counts (Model 3) or HIV Viral Load (Model 4). β values and p-values (in parentheses) are shown. Results are stratified by sample type (Plasma vs. PBMC), adjusting for year of sample collection. Time since infection was missing for 5 sequences, stage of infection for 242 sequences, CD4 count for 113 sequences, and viral load for 290 sequences with measured V1V2 length. Ref = Reference group. Analyses were performed for all sequences collectively as well as for sequences derived from plasma and PBMC considered separately. Sequences comprising the upper and lower 5% by length were excluded from these analyses. (0.06 MB PDF) [file ppat.1001228.s017.pdf]

Factor	Time since infection (Model 1)			Infection Stage (Model 2)		
	All seqs	Plasma	PBMC	All seqs	Plasma	PBMC
**Time since infection**	0.74 (0.8)	0.50 (<0.001)	0.72 (0.8)			
**Stage 1**				Ref	Ref	Ref
**Stage 2**				0.01 (0.9)	-0.01 (0.9)	0.00 (NA)
**Stage 3**				4.78 (<0.001)	5.23 (<0.001)	2.81 (0.2)
**Stage 4**				2.32 (0.07)	1.50 (0.18)	6.88 (0.02)
**Factor**	**CD4 count (Model 3)**			**Viral Load (Model 4)**		
**CD4 level**	**All seqs**	**Plasma**	**PBMC**	**All seqs**	**Plasma**	**PBMC**
**>500**	Ref	Ref	Ref			
**200-500**	0.41 (0.7)	0.32 (0.8)	6.36 (0.09)			
**<200**	-0.65 (0.7)	-1.34 (0.3)	5.12 (0.2)			
**VL log10 (continuous)**				-0.39 (0.3)	-0.30 (0.4)	-0.84 (0.15)

Table S2. Multivariable regression analysis of V1V2 length vs. clinical variables, upper and lower 5% excluded. Beta coefficients for V1V2 Length vs. Time since Infection (Model 1), Stage of Infection (Model 2), CD4 counts (Model 3) or HIV Viral Load (Model 4). β values and p-values (in parentheses) are shown. Results are stratified by sample type (Plasma vs. PBMC), adjusting for year of sample collection. Time since infection was missing for 5 sequences, stage of infection for 242 sequences, CD4 count for 113 sequences, and viral load for 290 sequences with measured V1V2 length. Ref = Reference group. Analyses were performed for all sequences collectively as well as for sequences derived from plasma and PBMC considered separately. Sequences comprising the upper and lower 5% by length were excluded from these analyses.
